# The potential of hydrogen hydrate as a future hydrogen storage medium

**DOI:** 10.1016/j.isci.2020.101907

**Published:** 2020-12-09

**Authors:** Ali Davoodabadi, Ashkan Mahmoudi, Hadi Ghasemi

**Affiliations:** 1 Department of Mechanical Engineering, University of Houston, 4726 Calhoun Road, Houston, TX 77204, USA

**Keywords:** Engineering, Mechanical Engineering, Materials Science, Energy Materials

## Abstract

Hydrogen is recognized as the “future fuel” and the most promising alternative of fossil fuels due to its remarkable properties including exceptionally high energy content per unit mass (142 MJ/kg), low mass density, and massive environmental and economical upsides. A wide spectrum of methods in ${\text{H}}_{2}$ production, especially carbon-free approaches, ${\text{H}}_{2}$purification, and ${\text{H}}_{2}$storage have been investigated to bring this energy source closer to the technological deployment. Hydrogen hydrates are among the most intriguing material paradigms for ${\text{H}}_{2}$storage due to their appealing properties such as low energy consumption for charge and discharge, safety, cost-effectiveness, and favorable environmental features. Here, we comprehensively discuss the progress in understanding of hydrogen clathrate hydrates with an emphasis on charging/discharging rate of ${\text{H}}_{2}$ (i.e. hydrate formation and dissociation rates) and the storage capacity. A thorough understanding on phase equilibrium of the hydrates and its variation through different materials is provided. The path toward ambient temperature and pressure hydrogen batteries with high storage capacity is elucidated. We suggest that the charging rate of ${\text{H}}_{2}$ in this storage medium and long cyclic performance are more immediate challenges than storage capacity for technological translation of this storage medium. This review and provided outlook establish a groundwork for further innovation on hydrogen hydrate systems for promising future of hydrogen fuel.

## Graphical Abstract

**Figure:**
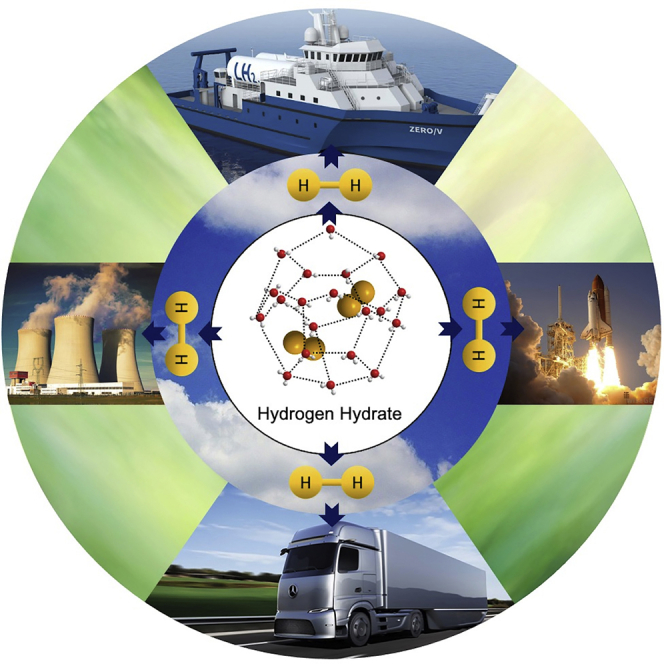

## Introduction

Energy availability is an absolutely essential factor for economic growth and plays a vital role in quality of human life. The exponential growth in world's population is accompanied with a substantial increase in energy demand: only from 2000 to 2019, the energy consumption has increased from 408 EJ to 585 EJ with a sustained increase of ca. 2%/year over the 2000–2018 period (Enerdata, 2017; Our World in Data, 2020). Currently, most of the energy demand (about 80%) is met by fossil fuels, e.g., oil, coal, and natural gas, leading to global concerns on climate change, air and water pollutions, and ozone layer depletion (Mahmoudi et al., 2018; Braungardt, van den Bergh and Dunlop, 2019; Khoshnevis Yazdi and Golestani Dariani, 2019; Kotcher et al., 2019). Therefore, the global search for possible alternative energy sources to replace the fossil fuels have been accelerated.

Solar energy, nuclear energy, wind energy, ocean wave energy, geothermal energy, and hydropower have been successfully employed for a variety of application (Khare et al., 2016; Liang, 2017); however, none of these energy sources can be directly used as a fuel for a wide range of applications such as land transportation, air transportation, and ocean-going vessels. Nonetheless, these sources can still be used to produce such a fuel (Veziroglu, 2007).

Hydrogen gas (${\text{H}}_{2}$) is known as a “green fuel” and has several remarkable properties that makes it the most promising option for fossil fuel replacement (Acar and Dincer, 2019): it is the chemically simplest and the lightest material and one of the most common elements on earth that has the highest energy content per unit mass (142 MJ/kg) compared with all other fuels (e.g., natural gas 53 MJ/kg) (Jain et al., 2010; Saadi et al., 2016).

Application of hydrogen has no negative impact on the environment, as hydrogen combustion only yields water vapor that has zero-emission feature (Haller and Link, 2017). Furthermore, due to its lower density compared to air and buoyancy effect, it dissipates quickly when it is released, allowing for relatively fast dispersal of the fuel in case of a leak (Kalinci et al., 2015).

Another promising incentive for hydrogen utilization is the ability to be used on the existing natural gas infrastructure of buildings, which gives it the capacity to address 10% of the global building heating demand by 2050. It is believed that widespread and full market penetration of renewable energy sources will be enabled through application of hydrogen. By 2050, hydrogen is projected to provide 18% of the final energy demand that can decrease 6 Gt/year ${\text{CO}}_{2}$ emission and provide about 30 million new jobs (Cipriani et al., 2014; Singh et al., 2016; Uyar and Beşikci, 2017). Also, statistical analysis predicts that by 2050, hydrogen will power over 5 million buses, 20 million trucks, and 400 million cars, which includes about 25% of the load of the transportation industry (Mostafaeipour et al., 2016). This is a significant shift in the grand scheme of energy consumptions, as currently 30% and 13% of total energy consumption at the end user is for transportation and residential applications, respectively (Gong et al., 2016; Valente et al., 2018). This transition requires three prerequisites to be fulfilled, i.e., the identification of the most appropriate hydrogen production source, hydrogen production technique, and hydrogen storage method.

Although some of hydrogen production techniques are carbon-free, other methods generate a mixture of ${\text{H}}_{2}$ and other gases such as $\;{\text{CO}}_{2}$, CO, and smaller quantities of methane (Sanchez-Hernandez et al., 2018). ${\text{H}}_{2}$ has to be separated from the mixture in order to be used as fuel, requiring an additional step after hydrogen production, i.e., hydrogen purification (Suri and Siddique, 2019; Chen et al., 2020). Furthermore, once pure hydrogen is obtained, it should be stored in a proper storage medium.

Hydrogen storage through hydrate formation is a relatively new technology that functions by enclathrating molecular ${\text{H}}_{2}$ inside the lattices of a crystalline host substance, i.e., water. Hydrogen clathrate hydrate is a promising medium for ${\text{H}}_{2}$ storage with immense benefits such as low energy consumption for charging and discharging, low fabrication costs, safety, and lack of negative environmental impact (Yu et al., 2020a; Wang et al., 2020). This study aims to review the latest developments in hydrate systems for hydrogen separation and storage and elucidate the open challenges in this research field.

## Hydrogen production and applications

**Figure 1:**
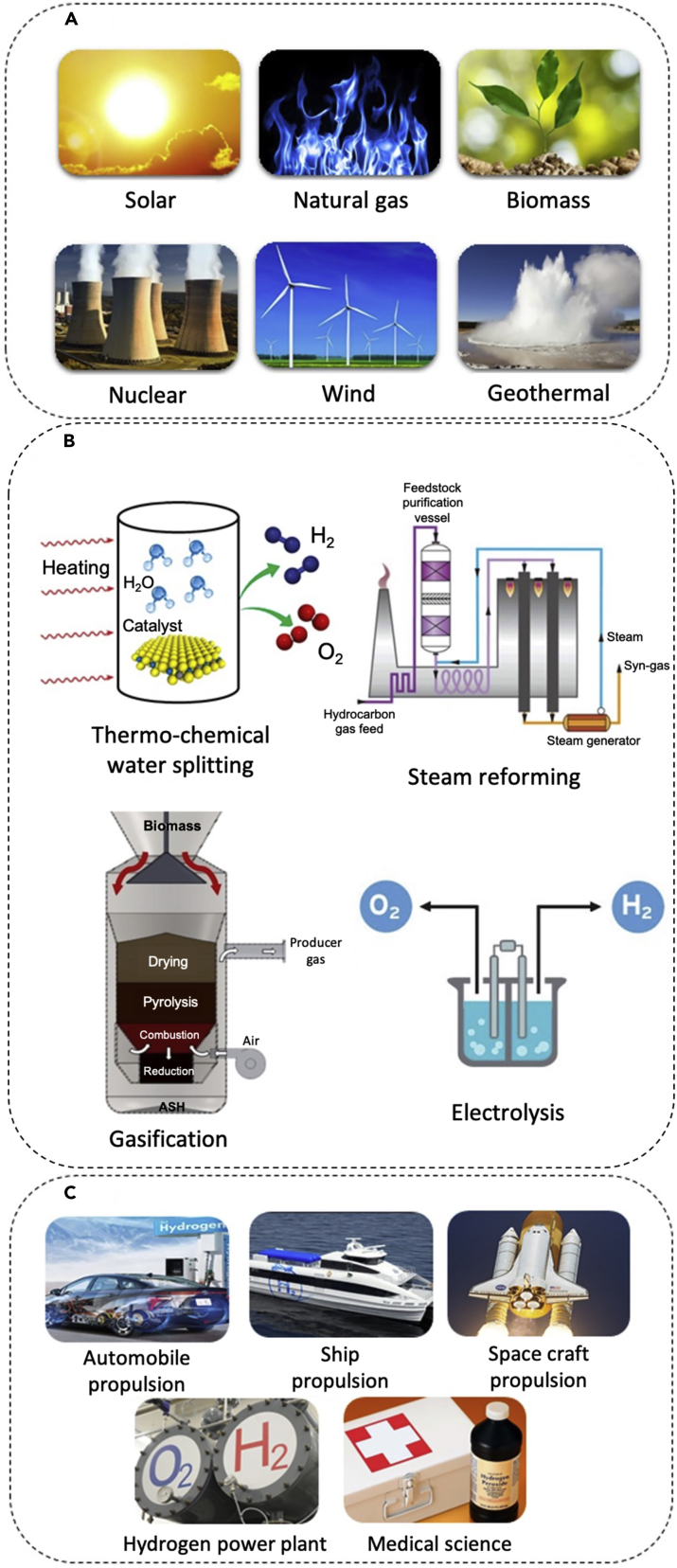
Hydrogen resources, production techniques, and applications The six major hydrogen sources, i.e., solar energy, natural gas, biomass, nuclear, wind, and geothermal energies (A). Source-dependent hydrogen production technologies: thermo-chemical water splitting for solar irradiation as the source, steam reforming for natural gas, gasification for biomass, and electrolysis for nuclear, wind, and geothermal energies (B). Gasification schematic adapted with permission from (De Lasa et al., 2011). The most prominent applications of hydrogen fuel include propulsion for automotive, ship and spacecraft, hydrogen power plants, and medical industry (C).

Solar energy, natural gas, biomass, wind energy, nuclear energy, and geothermal energy are promising sources for hydrogen (${\text{H}}_{2}$) production (Acar and Dincer, 2019) (Figure 1A), but each source requires a specific method for ${\text{H}}_{2}$ production. As shown in Figure 1B, thermo-chemical water splitting, methane steam reforming, gasification, and electrolysis are the most common hydrogen production methods (Agrafiotis et al., 2005; Palo et al., 2007; Gao et al., 2009). In thermo-chemical water splitting method, water reacts with a catalyst at a specific temperature, which depends on the catalyst material. A series of chemical reactions take place that produce hydrogen, oxygen, and release the catalyst (Kumar et al., 2018). These chemical reactions typically require elevated temperatures (from 573K to 923K) to operate effectively. Concentrated solar energy can be used to achieve the necessary temperatures for the reactions to occur (Han et al., 2007; James et al., 2007).

Methane steam reforming is another common process for hydrogen production, which uses methane as energy source and has a typical efficiency of 65%–75% (Liu et al., 2019b). In this method, the reaction between methane and high-pressure steam leads to production of hydrogen and carbon monoxide (CO), as shown in Reaction 1. Also, additional hydrogen can be extracted by steam reforming the generated carbon monoxide with water, where hydrogen and carbon dioxide (${\text{CO}}_{2}$) are the reaction products (Reaction 2) (Bruni et al., 2019; Leonzio, 2019).

(Reaction 1) $${\text{CH}}_{4}+{\text{H}}_{2}\text{O}\to 3{\text{H}}_{2}+\text{CO}$$

(Reaction 2) $$\text{CO}+{\text{H}}_{2}\text{O}\to {\text{ H}}_{2}+{\text{CO}}_{2}$$

In gasification method, biomass as the energy source reacts with steam to produce carbon monoxide and hydrogen (Reaction 3). Then through the reaction of carbon monoxide and water vapor, carbon dioxide and hydrogen are formed (Reaction 4) (Safarian et al., 2019; Chew et al., 2020).

(Reaction 3) $$\text{C}+{\text{H}}_{2}\text{O}\to {\text{ H}}_{2}+\text{CO}$$

(Reaction 4) $$\text{CO}+{\text{H}}_{2}\text{O}\to {\text{ H}}_{2}+{\text{CO}}_{2}$$

Electrolysis is another method that operates based on water splitting process, but rather than using heat and catalyst, electricity is used in this method for hydrogen production. Electrolysis setup consists of an anode and a cathode separated by electrolyte, wherein water reacts at the anode side to produce oxygen, positively charged hydrogen ions, and free electrons (Reaction 5). The electrons flow through an external circuit to the cathode side. At the cathode side, hydrogen is formed through reaction between the existing hydrogen cations and the electrons from the external circuit (Reaction 6) (Sapountzi et al., 2017; Chi and Yu, 2018).

(Reaction 5) $$2{\text{H}}_{2}\text{O}\to {\text{ O}}_{2}+4{\text{H}}^{+}+4{\text{e}}^{-}$$

(Reaction 6) $${4\text{H}}^{+}+4{\text{e}}^{-}\to 2{\text{H}}_{2}$$

The desirable cost for hydrogen production to make it a feasible fuel choice for industries is less than $2/kg (Reddi et al., 2017). Among different ${\text{H}}_{2}$ production methods, steam reforming and gasification techniques are capable to produce affordable hydrogen for less than $2/kg and therefore, supply more than 80% of the current hydrogen market. However, the major challenge in application of these methods is the co-production of ${\text{CO}}_{2}$ along with ${\text{H}}_{2}$. The two other methods, i.e., electrolysis and thermo-chemical water splitting, are carbon free but are more costly, with an approximate hydrogen production cost of about $3–5/kg. Thus, research and development (R&D) is focused on reducing the cost of hydrogen production through carbon-free methods (Dincer and Acar, 2014; Chi and Yu, 2018; Mohsin et al., 2018).

The produced hydrogen can be used as a fuel for a broad range of applications; as shown in Figure 1C. It can provide the required propulsion for land transportation sector (i.e. cars, bus, and truck) (Chilev and Lamari, 2016; Syed and Renganathan, 2019), ships in maritime industry (Smith and Fein, 2010; Nistor et al., 2018) as well as spacecraft propulsion (Frischauf, 2016) and power plant (Margiott et al., 2006). In particular, it can be supplied to fuel cells for electricity generation, running fuel cell-driven vehicles, ships etc. Hydrogen is also utilized in medical industry due to its capability to interact at the cellular level. Studies have indicated that hydrogen exerts antioxidant, anti-apoptotic, anti-inflammatory, and cytoprotective properties that are beneficial to cells (Dixon et al., 2013; Ge et al., 2017).

## Hydrogen purification

**Figure 2:**
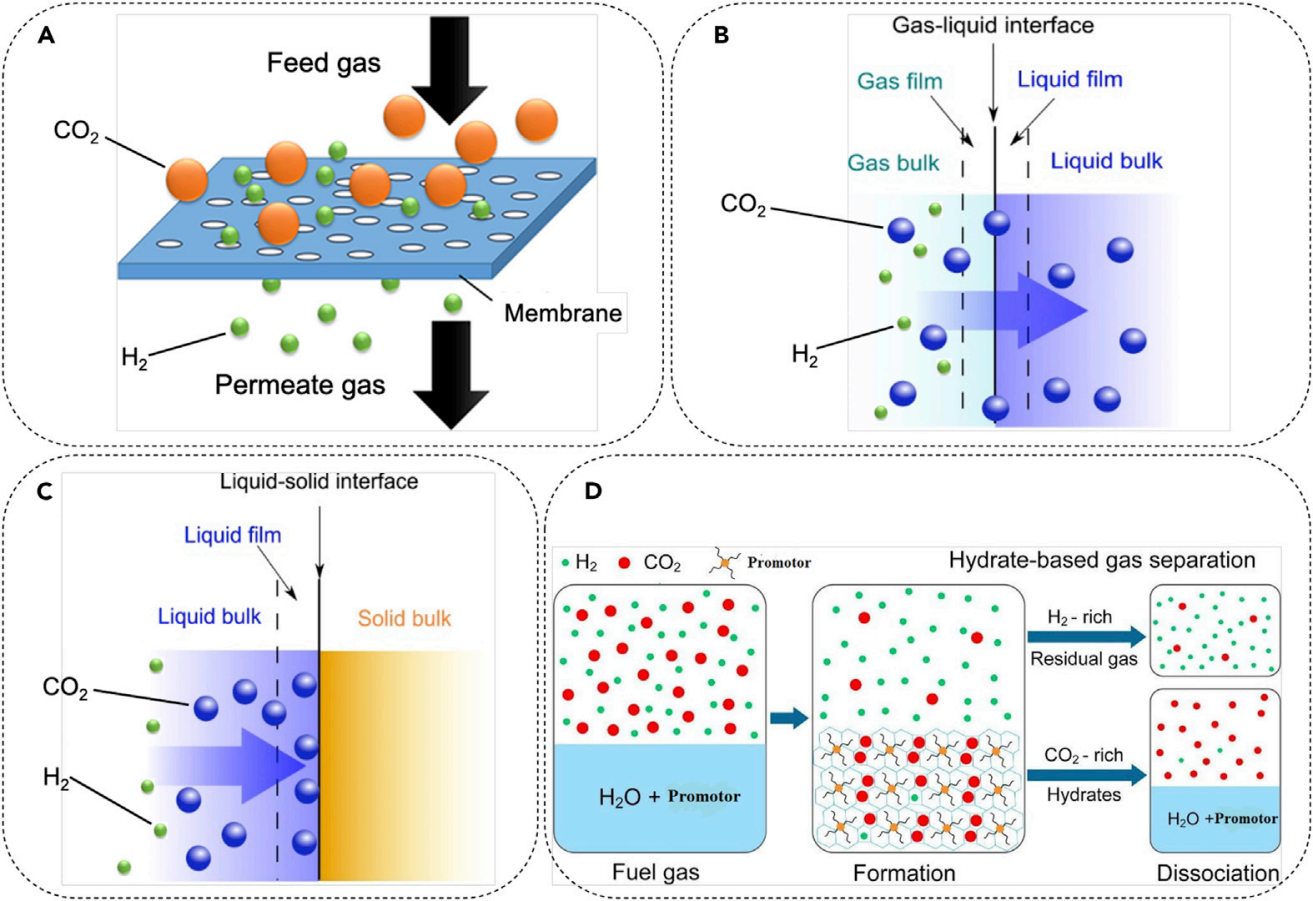
Hydrogen purification techniques Membrane method that selectively separates ${\text{CO}}_{2}$ molecules as the mixture passes through pores or small gaps in the molecular arrangement of a continuous structure (A), adapted form (Ji and Zhao, 2017). Absorption method by which ${\text{CO}}_{2}$ from the mixture is taken into the liquid phase so the species is separated from the mixture (B), adsorption method that involves separation of ${\text{CO}}_{2}$ from the mixture through its accumulation or concentration on a surface (C), carbon dioxide hydrate formation method that operates based on trapping the gaseous ${\text{CO}}_{2}$ molecule within a lattice cage created by the water molecules (D), adapted with permission from (Zheng et al., 2017).

As discussed previously, most of the current hydrogen production is through steam reforming and gasification methods. The final product of these methods is a combination of hydrogen, carbon dioxide, carbon monoxide, and small amounts of methane in some cases. To obtain pure ${\text{H}}_{2}$ that can be used as a fuel, the pollutant gases (and specially ${\text{CO}}_{2}$ that constitutes a large portion of them) need to be extracted from the mixture. As shown in Figure 2, four different techniques can be applied to obtain pure ${\text{H}}_{2}$ through ${\text{CO}}_{2}$/${\text{H}}_{2}$ separation: membrane separation (Zhao and Ho, 2013), absorption (Yan et al., 2011), adsorption (Belmabkhout and Sayari, 2009), and hydrate formation (Gholinezhad et al., 2011).

Membrane separation technology operates based on selective separation of molecules through pores in molecular arrangement of a continuous structure (Yu et al., 2015). This method has high energy efficiency because it does not require specific chemical processes or complex instrumentation and does not involve phase change during the separation process. In addition, it has low maintenance costs, as it does not have moving parts and complex controls. These properties make this method attractive for remote, unmanned, and footprint conscious sites (Liang et al., 2019; Saravanan et al., 2020). However, it suffers from issues including low purification efficiency. Also, the membranes are fabricated from expensive material and different studies have suggested the application of a stack of several membranes for the most efficient separation (Brunetti et al., 2010; Roy and Ragunath, 2018) that makes the assembly costly.

Absorption works based on the reaction between ${\text{CO}}_{2}\;$ and a chemical solvent such as aqueous solution of monoethanolamide (MEA), diethanolamine (DEA), di-isopropanolamine (DIPA), and methyldiethanolamine (MDEA). During this process, gaseous ${\text{CO}}_{2}\;$ passes through an amine solution until equilibrium is reached (Lapshin et al., 2020). The equilibrium condition varies for different solvents and is determined based on absorbing capacity of the solvent defined as the maximum molar amount of pollutant absorbed per mole of solvent (McCann et al., 2008). Even though studies have shown that the chemical absorption method enjoys a high purification efficiency, it has some shortcomings including solvent degradation, corrosion, and low solvent regeneration efficiency, which can affect the purification efficiency by time (Bernhardsen and Knuutila, 2017; Asif et al., 2018). Adsorption is another separation technology that is used for ${\text{H}}_{2}$purification. Separation is achieved through the significant intermolecular forces exerted on gas molecules from a solid surface. The effectiveness of this method depends on various parameters including temperature, partial pressure, surface force, and adsorbent pore sizes (Grande et al., 2008; Belmabkhout and Sayari, 2009). Zeolites, mesoporous silicates, alumina, metal oxide, activated carbons (ACs), and metal-organic frameworks (MOFs) are common materials that have been used for adsorption-based purification (Thiruvenkatachari et al., 2009; He et al., 2013). This technique is a dry process and has several advantages: it does not produce any by-product such as wastewater, and it requires less energy for regeneration compared with absorption processes. However, the drawbacks include low adsorption capacity of available adsorbents, lower purification efficiency compared with the absorption process, and limited reusability of the adsorbent (Riboldi and Bolland, 2017).

The other separation technique is the application of hydrate formation. This method works based on trapping the ${\text{CO}}_{2}$ molecules of the gas stream in the lattice of a crystalline material composed of water. Due to the high storage capacity of gas molecules in hydrate structures, this approach leads to efficient ${\text{CO}}_{2}\;$ removal. Nevertheless, the extremely high pressures and low temperatures required to form the carbon dioxide hydrate structure present a challenge for the application of hydrate formation as a separation technology. In addition, the hydrate formation rate is typically low, resulting in an economical barrier for implementation of this method. It should be noted that although it is possible to achieve the hydrate formation at moderate pressures and temperatures through the addition of a second guest molecule to the aqueous solution, this approach decreases the purification efficiency (Babu et al., 2015; Ma et al., 2016).

## Hydrogen storage

**Figure 3:**
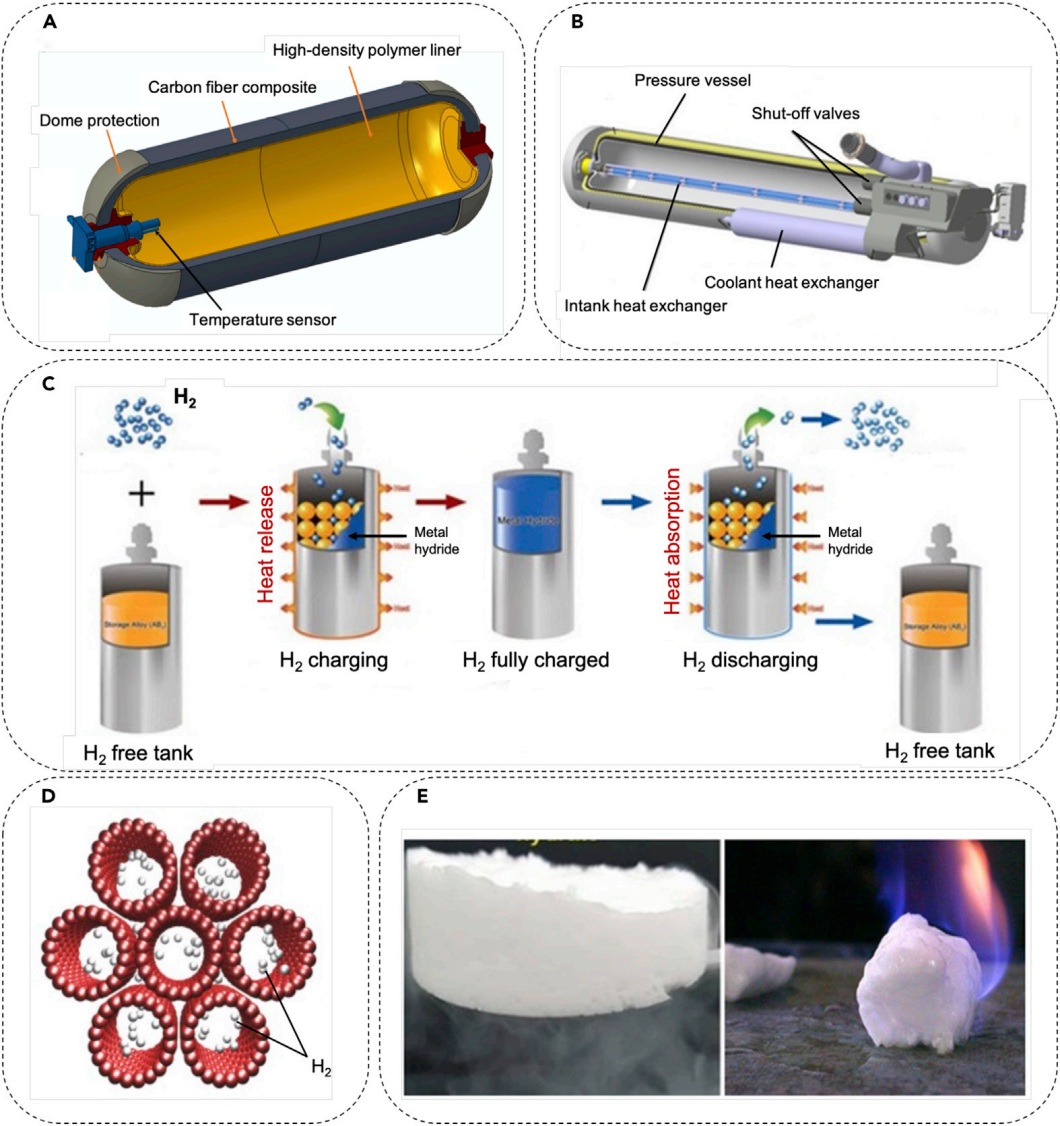
Hydrogen storage techniques High pressure tank whereby hydrogen gas is kept under high pressures to boost the storage density (A). Cryogenic tanks that are used to store hydrogen at cryogenic temperatures (B). Metal hydride technique that employs chemical reactions between certain metals and hydrogen to store hydrogen (C). Microporous carbon material with high surface area used to store hydrogen (D). Hydrogen hydrate formed based on physically trapping molecular hydrogen in water lattices (E).

A variety of technologies can be utilized to store hydrogen; the most common ones, i.e., high pressure gas tanks (Hua et al., 2011), cryo-compressed hydrogen storage (Yanxing et al., 2019), porous material (Xia et al., 2013; Sethia and Sayari, 2016), metal hydrides (Li et al., 2014; Tong et al., 2019), and hydrogen hydrates, are shown in Figure 3. These techniques employ different mechanisms and physical phenomena to store ${\text{H}}_{2}$ and, hence, offer different storage capacities. The underlying physics of storage mechanisms range from simple compression to liquefaction to absorption or even physically trapping the ${\text{H}}_{2}$ molecules. The storage capacities also depend on the respective physics; for example, in the trapping method, the strength of inter-molecular interactions such as covalent bonds and van der Waals interactions play a crucial role in medium's storage capacity (Zhou, 2005).

It must be mentioned that besides the storage capacity, the efficiency of the storage method is of critical significance. The efficiency of ${\text{H}}_{2}$ storage technologies is not merely a matter of mass or volume capacity; it is rather a measure of net stored energy. In other words, storage efficiency considers the required amount of energy for storing the gaseous ${\text{H}}_{2}$ and recovering it from the storage system as an additional important factor that must be taken note of besides the storage capacity (Di Profio et al., 2009). One of the desired properties of ${\text{H}}_{2}$ storage technologies is to require smaller amounts of volume to store ${\text{H}}_{2}$ in its thermodynamically stable state as a gas. This is particularly important as ${\text{H}}_{2}$ gas has very low density of only 0.089 $\text{kg}/{\text{m}}^{3}$, implying that very large volumes are needed to store a small mass of ${\text{H}}_{2}$. Notwithstanding, ${\text{H}}_{2}\;$ still serves as a promising fuel or energy carrier because it possesses a very high energy content by weight that can compensate its low density.

Application of high-pressure tank is one of the simplest and most common methods for hydrogen storage. The high-pressure tanks can accommodate a large amount of hydrogen in a small volume, are cost-effective, and relatively safe (Figure 3A). The major challenge for their application is the excessive pressures (typically 35–70 MPa) exerted by the compressed hydrogen, demanding the vessel material to be able to tolerate the potential embrittlement with hydrogen. Additional material qualifications include lightness and resistance against fire, high temperatures, and degradation. Inexpensiveness and easy handling for transport and online safety monitoring are among other important characteristics (Jorgensen, 2011; Liu et al., 2012; Dagdougui and Ouammi, 2018).

Cryogenic storage method operates based on liquefying the hydrogen by cooling it to 20 K (Figure 3B), which enhances the volumetric energy density; therefore, less volume is required for storage and a smaller, lighter container can be used. The major problem with this method is the associated cost. In fact, about 35% of energy content of the fuel is used for liquefaction, which implies a three-fold increase in energy consumption compared with compression of ${\text{H}}_{2}$ to 70 MPa (Arnold and Wolf, 2005; Zohuri and Zohuri, 2019).

Application of metal hydrides is one of the most compact approaches to store ${\text{H}}_{2}$. It consists of containers that are filled with a metal that is capable of absorbing and discharging ${\text{H}}_{2}$, as shown in Figure 3C. There are two types of metal hydrides: binary hydrides and intermetallic hydrides. Binary hydrides contain only one metal with a formula of ${\text{MH}}_{\text{x}}$ where M represents the metal, whereas intermetallic hydrides contain more than one metal with a formula of ${\text{A}}_{\text{m}}{\text{H}}_{\text{x}}{\text{B}}_{\text{n}}$ where A and B represent the metals (Luo et al., 2018). Intermetallic hydrides are more common compared with binary ones mainly due to their higher gravimetric capacity and capability to operate at pressures and temperatures closer to ambient conditions. Despite their advantageous properties such as their compactness, the expenses incurred by high alloy costs required for their operation remain as a major drawback for the application of metal hydrides (Jain et al., 2010; Ward et al., 2016; Stamatakis et al., 2018). In addition, the chemisorption of ${\text{H}}_{2}$ into metallic surfaces that is typically associated with large binding energies requires elevated pressures. Also, the subsequent ${\text{H}}_{2}\;$ compounds formation generates heat as a result of the exothermic reactions that take place during adsorption (Chaudhuri and Muckerman, 2005). Thus, to release the stored ${\text{H}}_{2}$ (i.e., to reverse the adsorption process), pressure must be decreased, and heat needs to be applied to the system—two requirements that are not, particularly, desirable. Nevertheless, it must be mentioned that some metal hydrides can release ${\text{H}}_{2}$ near ambient temperatures; but they suffer from very low gravimetric hydrogen storage capacities (below 3 wt.%) (Sakintuna et al., 2007; Jorgensen, 2011). Although there exist some light metal hydrides and complex hydrides with high gravimetric storage capacities (e.g., 7.6 wt.% for ${\text{MgH}}_{2}$ and 18 wt.% for ${\text{LiBH}}_{4}$), elevated temperatures are needed to release ${\text{H}}_{2}$ from these hydrides. Moreover, the slow kinetics of dehydrogenation is yet another major challenge for application of metal hydrides for ${\text{H}}_{2}$ storage (Vajo et al., 2007).

Porous media such as zeolites, porous carbon structures, and metal organic frameworks (MOFs) can also be used as another ${\text{H}}_{2}$ storage medium, where the physisorption of ${\text{H}}_{2}$ on the surface of the pores of the material, as shown in Figure 3D, allows for ${\text{H}}_{2}$ storage. Surface area, pore size, and pore volume are the most important parameters determining the storage capacity of these porous media. In addition, different studies have indicated that hydrogen adsorption of microporous storage material enhances with decreasing the temperature and/or increasing the pressure. A porous material with large pore volume and narrow pore size range results in large hydrogen uptakes. On the other hand, a decrement in temperature that increases the absorption capability of the porous material can make the micropore size distribution less important (Jordá-Beneyto et al., 2007; Xia et al., 2013; Berenguer-Murcia et al., 2018; Mohan et al., 2019).

Even though, many of the physisorption-based materials have acceptable ${\text{H}}_{2}$ storage capacities at low temperature (∼77 K) and high pressure conditions, but at ambient temperature and in a pressure range of 1–50 bar, their capacity decreases to lower that 1 wt.% (Mananghaya, 2019), implying that low temperatures are, indeed, essential to their application. On the other hand, storing ${\text{H}}_{2}$ at low temperatures (e.g., ∼77 K) may not be economically rational. For example, consider the binding energy of ${\text{H}}_{2}$ onto a certain surface to be ∼10 kJ/mol; hence, adsorption of 6 kg of ${\text{H}}_{2}\;$ will generate 30 MJ heat. If liquid ${\text{N}}_{2}$ with a heat of vaporization of ∼5.6 kJ/mol is used to maintain ${\text{H}}_{2}$ at such low temperatures, a simple calculation yields that 5400 mol (i.e., ∼150 kg) liquid ${\text{N}}_{2}$ would be required to circulate around the cylinder to maintain that temperature (Oelerich et al., 2001). Such a large amount of liquid ${\text{N}}_{2}$ for cooling the storage system introduces further difficulties for the engineering design as well (Contescu et al., 2009; Heo et al., 2019).

In this paper, we focus on hydrate-based media for storage of hydrogen. Hydrates are crystalline materials consisting of lattices of one compound (mostly water molecules) that trap a second compound.

Compared with other storage methods, hydrate formation method offers great advantages. Firstly, unlike metal hydrides, the decomposition kinetics can be very fast and very small amount of energy is required for decomposition of the hydrate lattice (i.e. heat of fusion). In fact, hydrogen can be readily released by increasing the temperature or lowering the pressure of the hydrate system. Secondly, it is a very inexpensive technology with high level of safety and absence of negative environmental impact. Indeed, the main component of the hydrate system, i.e., water, is abundant and cheap. It is neither flammable nor corrosive and do not generate any pollutant/toxic substance. In addition, it can retain a significant amount of hydrogen (∼5 mass % molecular hydrogen), and disposal costs are very insignificant, which makes it even more cost-effective. Figure 3E shows the potential of hydrogen hydrate to sustain a flame.

However, the major challenge with this technique is the excessively high pressures required for its operation. Pressure as high as several hundred MPa (e.g., 200 MPa at 273K) is typically necessary for hydrogen hydrate formation. Notwithstanding, studies have shown that inclusion of a second guest molecule, namely a promotor, is useful to form and store hydrogen at lower pressures, whereas it compromises the hydrogen storage capacity of the hydrate (Di Profio et al., 2009; Chattaraj et al., 2011; Ozaki et al., 2014; Veluswamy et al., 2014b). On the other hand, in this technique, hydrate formation is governed by diffusion through a bulk solid phase. This results in relatively slow rates of hydrate formation that poses as another major challenge for this storage method. For example, the slow kinetics of hydrogen hydrate formation imposes difficulties for onboard recharging of ${\text{H}}_{2}\;$ for fuel cell-driven vehicles (Lang et al., 2010). Continuous cooling of the system, which is necessary to retain the stability of hydrogen hydrates at low pressures, is another challenge for application of these material. In addition, any accidental failure in the cooling system can result in serious safety issues, as the hydrate structure can release large amounts of hydrogen in a relatively short time (Hu and Ruckenstein, 2006).

## Hydrogen hydrate

### Discovery

After the discovery of naturally occurring hydrate deposits, interest has surged among the researcher to understand the energy and environmental impact of these hydrate deposits (Rempel and Buffett, 1997; Zatsepina and Buffett, 1997). Over the past decades, the gas hydrate formation phenomenon has been applied to develop technologies for natural gas storage, carbon dioxide capture, gas separations, and hydrogen storage (Cha et al., 2010). Among different gas hydrates, hydrogen hydrate, in particular, has been notorious to be extremely hard to form, primarily due to the small molecular size of hydrogen. It was only in 1990s when Dyadin et al. (Dyadin et al., 1999), who were studying the ${\text{H}}_{2}\text{O}$–${\text{H}}_{2}$ system, realized that when ${\text{H}}_{2}\;$ pressure is raised above 100 MPa, a temperature region exhibiting anomalous melting behavior and kinetics of ice melting is observed. They attributed this observation to the formation of hydrogen hydrate in this pressure range. These studies were followed by additional investigations that elaborated on the original findings and discussed the details of hydrate formation (Mao et al., 2002; Lokshin et al., 2004).

Despite the success in forming hydrogen hydrate, it was soon realized that the extreme pressure/temperature conditions required for hydrogen hydrate formation may not be accessible for many potential applications. Hence, subsequent studies focused on moderating the required conditions for ${\text{H}}_{2}$ storage through hydrate formation process. For example, it was shown that addition of tetrahydrofuran (THF) to water can decrease the hydrogen hydrate formation pressure from 200 MPa to 100 MPa at ambient temperature. Zhang et al. (Zhang et al., 2005) also showed that the required pressure for formation of the binary ${\text{H}}_{2}/{\text{CH}}_{4}$ hydrate can be drastically lowered from 6.5 MPa to 0.3 MPa by adding small concentrations (6 mol.%) of THF to water. A variety of such additives known as promotors have been used in different studies to facilitate hydrate formation by lowering the required pressure. Another important landmark in this field was the introduction of semi-clathrate hydrates for hydrogen storage. Indeed, although the addition of promotors, e.g., THF, can moderate the required conditions for hydrate formation, the necessary pressure in the order of tens of MPa may still not be reachable for some applications. Semi-clathrate hydrates could be an alternative option in such circumstances. Chapoy et al. (Chapoy et al., 2007) developed semi-clathrate structures for hydrogen storage that were stable at atmospheric pressure and room temperatures. They, successfully, stored hydrogen in the structural cavities of semi-clathrate hydrates of quaternary ammonium salts (QAS). These hydrates demonstrated stability at atmospheric pressure and up to ∼30°C temperatures. This is primarily due to the guest molecules physically bonding with the water structure as well as filling in the cavities.

### Hydrogen storage mechanism in hydrate

The mechanism of ${\text{H}}_{2}$ storage through hydrate formation is similar but not identical to physisorption mechanism. The storage of ${\text{H}}_{2}$ molecules in hydrate structure occurs by physically capturing the ${\text{H}}_{2}$ molecules in ${\text{H}}_{2}\text{O}$ cages as opposed to chemical reaction or adsorption. The mechanism is primarily governed by van der Waals (dispersion) forces, intermolecular interactions, and hydrogen bonding, where the strong hydrogen bonds hold the host framework together (Struzhkin et al., 2007). Once water and ${\text{H}}_{2}$ are mixed, the guest molecules incorporate into the polyhedral cages of the host framework and form hydrogen hydrate, a process that typically requires low temperatures and elevated pressures to take place (Mao et al., 2002). The guest molecules can be placed in large cages, small cages, or in both, based on their size. Mao et al. (Mao et al., 2002) investigated hydrogen hydrate formation, which was synthesized as a liquid at pressure of 200 MPa and temperature of −24°C. Their results indicated that four and two ${\text{H}}_{2}$ molecules were stored in a large and a small hydrate cage, respectively. In another study, Lokshin et al. (Lokshin et al., 2004) used neutron diffraction to investigate the composition of hydrogen hydrate phase. It must be noted that the background signal generated from incoherent scattering of hydrogen makes detection of the Bragg scattering from ${\text{H}}_{2}-{\text{H}}_{2}\text{O}$ systems very challenging. Hence, alternative hydrogen hydrate systems containing deuterated water (${\text{D}}_{2}\text{O}$) and ${\text{D}}_{2}$, such as ${\text{D}}_{2}-{\text{H}}_{2}\text{O}$, $\text{HD}-{\text{H}}_{2}\text{O}$, and ${\text{H}}_{2}-{\text{D}}_{2}\text{O}$ systems, are adopted for such studies. The neutron diffraction results obtained by Lokshin et al. indicated that ${\text{D}}_{2}$ occupancy in the large cages can change between two to four molecules per cage by variation of pressure and/or temperature. However, the occupancy of the small cages was constant and equal to one molecule, nearly up to the decomposition temperature of the hydrate. These occupancy numbers reflect the average number of molecular hydrogens in small and large cages. In fact, a single molecule of hydrogen is too small to stabilize a small cage with 7Å characteristic length. Hence, clathrate cages are often host to clusters of hydrogen molecules. In a recent study, Li et al. (Li et al., 2018) demonstrated that clusters containing up to four hydrogen molecules can be stored in a small clathrate cage, whereas large cages can store more hydrogen molecules. Such observations have been used to infer the stoichiometry of hydrogen occupancy in the clathrate hydrate and estimate the molar ratio of hydrogen to water in hydrate structures. For example, it has been shown that hydrogen occupancy can vary from 32${\text{D}}_{2}$.136${\text{D}}_{2}\text{O}$ to 48${\text{D}}_{2}$.136${\text{D}}_{2}\text{O}$, in simple hydrogen hydrates. It, also, can be written as 32(1 + x)${\text{D}}_{2}$.136${\text{D}}_{2}\text{O}$ where x varies between 0 and 16 depending on pressure and temperature. The corresponding ${\text{D}}_{2}$/${\text{D}}_{2}\text{O}$ molar ratio varies from 0.26 to 0.35 (Lokshin et al., 2004).

On the other hand, the stability of the hydrogen hydrate structure relies on the existence of guest molecules inside the large cages. In fact, the dispersive interactions between ${\text{H}}_{2}$ and water molecules that form the cage walls is a deterministic factor for the stability of the hydrogen hydrate structure (Patchkovskii and Tse, 2003). Without the support of the trapped ${\text{H}}_{2}$ guest molecules, the hydrate structure would collapse into liquid water. While, it is not necessary to have 100% occupation of the large cages to achieve a stable hydrate structure (Katsumasa et al., 2007), it is quite common for the large cages to be filled to about 100% occupancy. For the small cages the occupation range can vary from 0% to 100% (Strobel et al., 2007a). Raman spectroscopy investigations have shown that the roton peaks for ${\text{H}}_{2}$ in the hydrate were identical to those of pure ${\text{H}}_{2}$ In other words, ${\text{H}}_{2}$ molecules demonstrated free rotations inside the hydrate cages, implying that the ${\text{H}}_{2}$ molecules in the hydrate cages were still in free rotational states. This observation can be further interpreted, as the ${\text{H}}_{2}$ molecules stored in the hydrate structure remain unbonded to one another, as well as to water molecules (Mao et al., 2002). Based on the size of the guest molecules, three different types of hydrate structures, i.e., cubic structure I (sI), cubic structure II (sII), and hexagonal cubic structure (sH), can form (Koh, 2002). The storage capacity of ${\text{H}}_{2}\;$ in hydrate structures is determined based on the size and the structural type of a clathrate hydrate (Chattaraj et al., 2011).

Application of memory water, i.e., water previously utilized for hydrate formation and restored after hydrate dissociation, and addition of a second guest molecule (i.e., a promotor) are two additional important techniques that can affect the mechanism of and the condition for hydrogen hydrate formation (Rasoolzadeh and Shariati, 2019). The clusters of water molecules, which were arranged as part of the hydrate structure, would remain stable even after hydrate dissociation. These clusters of water molecules, present in memory water, considerably facilitate the (re)formation of hydrate nuclei—a property that is primarily due to the remaining bonding water structure in the memory system. The stability of these clusters depends on the pressure and temperature of the system, where the stability increases as the pressure increases, but it goes down with superheating of water above the hydrate equilibrium point after hydrate decomposition (Patchkovskii and Tse, 2003; Lee et al., 2010).

Addition of promotor molecule also affects hydrogen hydrate formation mechanism. Even though using second guest molecule decreases the storage capacity by occupying portions of the available room especially in larger cages, it can enhance hydrogen hydrate nucleation through ${\text{H}}_{2}$ adsorption onto the second guest molecule surface. These ${\text{H}}_{2}$ molecules subsequently diffuse into the hydrate structure (Nagai et al., 2008). In addition, promotor can significantly facilitate hydrate formation by lowering the required pressure. For example, addition of THF to ${\text{H}}_{2}$–${\text{H}}_{2}\text{O}$ system has been shown to reduce the formation pressure by a factor of 30 (from 200MPa to 7MPa at 280K) (Florusse, 2004; Lee et al., 2010).

### Hydrogen hydrate structures

**Figure 4:**
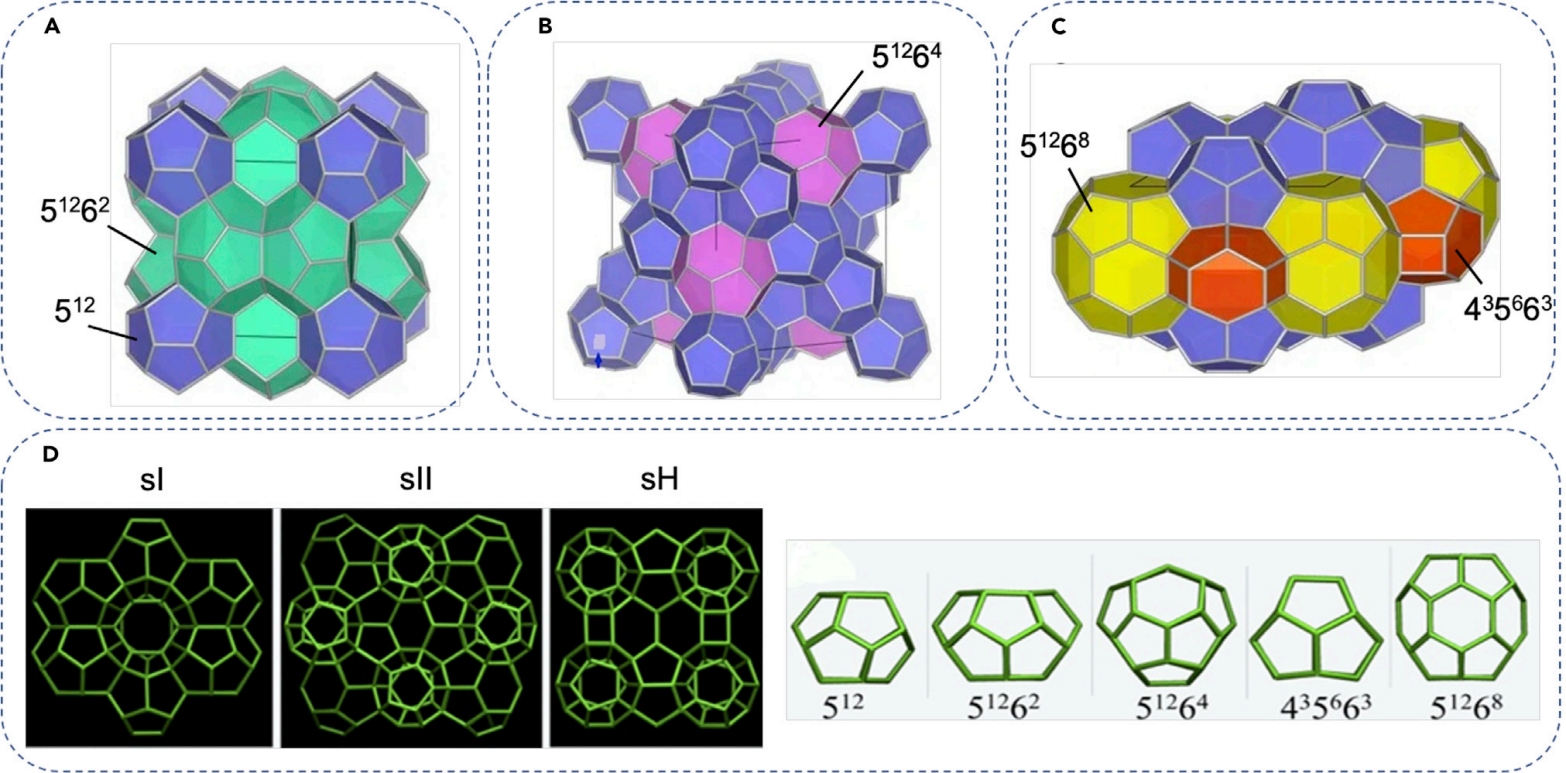
Hydrogen hydrate structures 3D structure of sI hydrate: the unit cell consists of 46 water molecules arranged into small cages with twelve pentagonal faces and large cages with two hexagonal and twelve pentagonal faces (A). 3D structure of sII hydrate with the unit cell composed of 136 water molecules arranged into small cages with twelve pentagonal faces and large cages with twelve pentagonal and four hexagonal faces (B). 3D structure of sH hydrate with the unit cell composed of 34 water molecules arranged into small cages with twelve pentagonal faces; medium cages with three hexagonal, six pentagonal, and three tetrahedral faces; and large cages with twelve pentagonal and eight hexagonal faces (C). (A), (B), and (C) are adapted with permission from (Momma et al., 2011). 2D view of hydrate structures and building blocks including small, medium, and large cages (D), adapted with permission from (Liang and Kusalik, 2015).

Clathrate hydrates are a special type of inclusion compounds with solid cage-like crystalline structures that physically resemble ice and are formed by pentagonally or hexagonally hydrogen-bonded water molecules. These cages trap the guest molecules by developing van der Waals interactions between the guest molecules and the surrounding water cage walls. Figure 4 shows 2-dimensional (2D) and 3-dimensional (3D) representations of the three possible hydrogen clathrate structures, i.e., sI, sII, and sH, where the sH type is far less common than the formers (Momma et al., 2011; Liang and Kusalik, 2015). In all clathrate structures, water molecules and hydrogen bonds constitute the vertices and the edges, respectively; however, each structure has its own crystallographic properties and contains geometrically specific water blocks with various cage shapes and sizes. sI structure contains 46 water molecules that form 8 cages per unit cell (Izquierdo-Ruiz et al., 2016): two smaller cages that are made up of 20 water molecules and are arranged in a stretched pentagonal dodecahedron configuration with $5^{12}$ faces and six larger hexagonal truncated trapezohedron cages made up of 26 water molecules with $5^{12}6^{2}$ faces (Figure 4A), where ${\text{X}}^{\text{m}}{\text{Z}}^{\text{n}}\;$ represents a cage with m X-sided and n Z-sided faces (Willow and Xantheas, 2012; de Menezes et al., 2019). sII hydrates, on the other hand, contain 136 water molecules and are made up of sixteen small $5^{12}$ cages and eight larger $5^{12}6^{4}$ cages in a unit cell (Klapproth et al., 2019). Lastly, sH hydrates contain 36 water molecules and include three $5^{12}$ cages and two irregular dodecahedrons $4^{3}5^{6}6^{3}$ cages and one icosahedron large $5^{12}6^{8}$ cage in a unit (Khokhar et al., 1998; Chattaraj et al., 2011; Lee et al., 2016). The thermodynamic stability of the hydrate is quantified by the cohesive energy$\;E_{coh}$, which is the difference between the value of total energy of the separated monomer molecules and the energy of the hydrate:

(Equation 1) $$E_{coh}=\frac{\left(x\;.\;E_{hydrogen}+y\;.\;E_{water}+z\;.\;E_{promotor}\;\right)-E_{hydrate}}{x+y+z}$$

In Equation 1, $E_{hydrogen}$, $E_{water}$,$\;E_{promotor}$, and $E_{hydrate}$ denote the energies of the hydrogen molecule, the water molecule, the promotor molecule, and the hydrate, respectively. Also, x, y, and z represent the number of the hydrogen molecules, water molecules, and the promotor molecules, respectively (Liu et al., 2017; Zhong et al., 2020). The stability and the binding strength of the hydrogen molecules to the hydrate is computed by the interaction energy, $E_{int}$:

(Equation 2) $$\;E_{\mathrm{int}}=\left(\;E_{hydrogen}+\;E_{residue}\right)-\;E_{hydrate}$$

where $E_{residue}$ indicates the energy of the hydrate with a lost hydrogen molecule (Liu et al., 2019a).

The preferred structure of the clathrate is determined by the configuration that stabilizes the mixture by achieving the best match between the size of the guest molecule and the composition to the size and composition of the cages. Matsumoto and Tanaka (Matsumoto and Tanaka, 2011) drew a phase diagram for hydrate formation by plotting the chemical potential of water against the Lennard-Jones parameters for a single component gas in different hydrate structures. The results indicated that, in agreement with previous observations (Sloan, 1998; Jacobson et al., 2009; Jacobson and Molinero, 2010), as the guest molecule size increases, the crystal structure changes from sII to sI, then partially occupied sI takes over, and finally partially occupied sII structure recovers upon further increase in molecule size. Their theoretical predictions, also, envisioned the possibility for emergence of sH clathrate when the interactions between guest and host are very strong. However, the sH structure is very rarely formed, as it may become the stable phase only around the phase boundary between sI and sII where the chemical potentials of the two phases compete.

Although it might be expected to observe alternative hydrate structures for multicomponent gases, simulations have predicted that sI and sII clathrates will still remain the only dominant structures that form when more than one gas is involved in hydrate formation (Matsumoto and Tanaka, 2011). Nevertheless, it is noteworthy that a clathrate hydrate of gas mixture sometimes forms a crystal structure different from the crystal structure that each gas prefers. For example, the gas mixture of methane and ethane at a specific composition forms sII structure, whereas methane and ethane both form sI hydrates, individually (Koyama et al., 2005).

It is worthy to note that at extremely elevated pressures (i.e., tens to hundreds of GPa), hydrate structures transition from clathrate to filled ice structure. However, because hydrate systems for hydrogen storage at moderate pressures are of interest of this review, we limit our discussion to clathrate structures.

### Hydrogen hydrate formation

#### Phase equilibrium data

**Figure 5:**
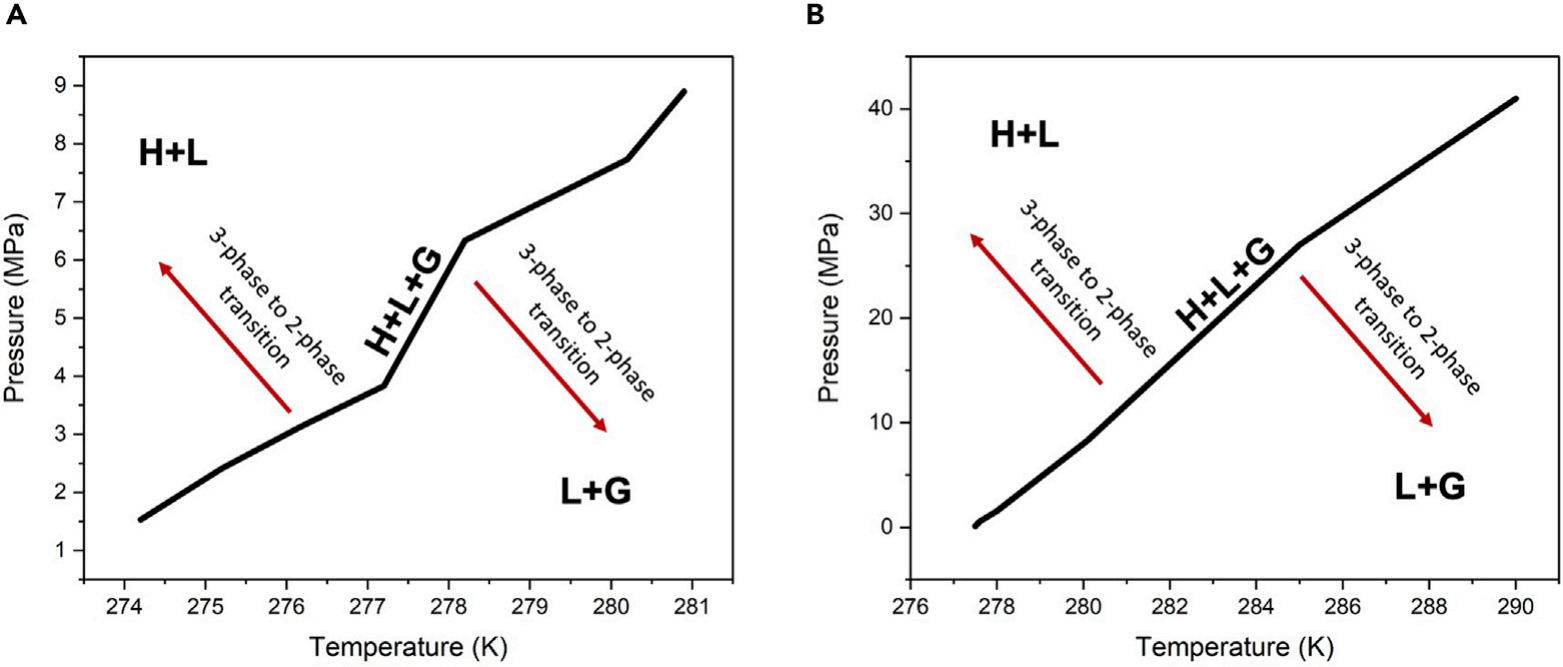
Hydrogen hydrate phase diagrams Phase diagram of hydrogen hydrate structure with TBAB as the promotor (A) and phase diagram of hydrogen hydrate structure with THF as the promotor (B). H, L, and G represent the hydrate, liquid, and gas phases, respectively. Note that hydrate is only stable in the regions above the three-phase equilibrium line.

Hydrate structure can be considered as a hydrogen battery that can switch between charging and discharging states depending on the enforced conditions. The specific condition is defined as the phase equilibrium condition. Phase equilibrium data provide the conditions for coexistence of three phases of gas, liquid, and hydrate. For instance, Figure 5A and b show the phase diagrams of the hydrogen hydrate with TBAB and THF as the promotor, respectively. H, L, and G represent the hydrate, liquid, and gas phases, respectively. As can be seen, by increasing the pressure and/or decreasing the temperature at equilibrium condition, the three-phase mixture including hydrate, gas, and liquid transforms into two-phase mixture including hydrate and liquid. The required time for this transition depends on the formation rate (i.e. charging rate). On the other hand, a deviation from the equilibrium condition due to a decrease in pressure and/or increase in temperature drives the system into hydrogen discharge state where the three-phase mixture transforms into a two-phase mixture including liquid and gas. Table S1 in the Supplemental Information shows the phase equilibrium data of different hydrogen hydrates reported in the literature. Smirnov et al. (Smirnov and Stegailov, 2013) studied hydrate formation of pure hydrogen over a range of pressures and temperatures. The results indicated that hydrogen formation without promotors occurs at very high pressures and/or very low temperatures. In order to be able to form hydrates at near ambient temperatures, pressure of 200 MPa is required. Studies have demonstrated that the addition of promotors can significantly improve the chances of hydrate formation at lower pressures and/or higher temperatures. Du et al. (Du et al., 2011, Du et al., 2012) observed hydrogen hydrate formation at ambient pressure by using tetrahydrofuran (THF), tetra-n-butylammonium bromide (TBAB), and tetrabutylphosphonium bromide (TBPB) as promotors. Komatsu et al. (Komatsu et al., 2010) showed that 4° increase in the temperature from 278K to 282K results in a jump in the required pressure for hydrate formation from 2 MPa to 12 MPa. In another study, Karimi. et al. (Karimi et al., 2014) employed tetrabutylammonium hydroxide (TBAOH) as the promotor and found that as the temperature increases from 286K to 290K, the required pressure for hydrate formation increases from 1MPa to 20 MPa. The reported values for different pressures and temperature show that the operating temperature is of paramount importance for successful formation of hydrogen hydrate.

Because promotors have shown a promising prospect of facilitating the hydrate formation process, studies have focused on comparing different promotors with different hydrate equilibrium conditions. Hashimoto et al. (Hashimoto et al., 2008) compared equilibrium condition of two different promotors, i.e., THF and TBAB. The results indicated the superiority of TBAB for hydrate formation: when TBAB was used as the promoter, they were able to form hydrate at 286 K and 6 MPa, whereas, with THF, hydrate formed at 280 K and 8.3 MPa. Hence, hydrate can form at higher temperatures and lower pressures if TBAB is employed as the promoter. In another study, by comparing the performance of two different promotors, i.e., tetrahydrothiophene (THT) and furan, Tsuda et al. (Tsuda et al., 2009) concluded that furan is preferred over THT as the promotor. The results indicated that with 0.05 molar concentration of promotors and at an identical temperature of 277 K, hydrogen hydrate forms at 0.11 MPa with furan, whereas an extremely higher pressure of 5.86 MPa is needed when THT is used as the promoter. Martín et al. (Martín and Peters, 2009) investigated the required temperatures and pressures for coexistence of three phases, including liquid, gas, and hydrate, while using methyl tert-butyl ether (MTBE) and 1,1-dimethyl cyclohexane (DMCH) as promotors. DMCH was identified as the preferred choice for promoter because higher pressures were needed to form hydrate with MTBE. However, it must be noted that compared with other promotors discussed in this paper, both DMCH and MTBE require considerably higher pressures for hydrate formation. Zhdanov et al. (Zhdanov et al., 2017) chose argon (Ar) as the promotor and studied its effect of on hydrate equilibrium condition. Hydrate formation at lower pressures compared with pure hydrogen, was successfully achieved; however, the required pressures were significantly greater than those needed with other promotors. For example, a pressure of ca., 32 MPa was required to form hydrate at a temperature of 200 K, whereas, hydrate can form at pressures as low as 4 MPa and 8 MPa with TBAB and THF, respectively.

A combination of promotors can be used to facilitate the hydrate formation process as well. Florusse et al. (Florusse, 2004) and Zhang et al. (Zhang et al., 2005) compared the equilibrium state for two different cases: one with THF as the only promotor and one using a combination of ${\text{CH}}_{4}/\text{THF}$. They found that ${\text{CH}}_{4}/\text{THF}$ combination leads to hydrate formation at more moderate conditions compared with the case where only THF was used. They further demonstrated that with a fixed concentration of THF, increasing the amount of ${\text{CH}}_{4}$ lowers the required pressure for hydrate formation. That is, at the temperature of 277.7 K, with 0.65 mol and 0.21 mol of ${\text{CH}}_{4}$, hydrate formed at 0.2 MPa and 0.55 MPa, respectively. The appropriate choice of promotors combination is critical for hydrate formation. Khan et al. (Khan et al., 2015) studied hydrate equilibrium condition by considering two systems with different combined promotors: one using a mixture of$\;{\text{CH}}_{4}$ and methylcyclohexane and the other using a mixture of ${\text{CH}}_{4}$ and THF as the promotors. The results suggested that the latter requires lower pressures for hydrate formation than the former and, hence, is preferable.

In order to understand the combined effect of promoter and temperature on hydrate formation, Fujisawa et al. (Fujisawa et al., 2012) studied the variation in hydrate equilibrium data with temperature while using TBPB as the promotor. They observed that small variations in the temperature changes the pressure drastically. For example, increasing the temperature from 282 K to 296 K raised the required pressure from 0.11 to 164 MPa. These results further highlight the importance of temperature in determining the hydrogen hydrate formation condition.

Composite ${\text{CO}}_{2}/{\text{H}}_{2}\;$ is an alternative to pure hydrogen hydrate systems for hydrogen storage. In fact, application of ${\text{H}}_{2}\text{O}-{\text{CO}}_{2}/{\text{H}}_{2}$ hydrate obviates the need for further ${\text{CO}}_{2}$ separation as a common by-product in hydrogen production (see Reactions 2 and 4). Studies on the role of promotors in composite ${\text{H}}_{2}\text{O}-{\text{CO}}_{2}/{\text{H}}_{2}$ hydrate systems indicate analogous trends as those in pure hydrogen hydrate systems. In order to investigate the effect of promotor concentration on equilibrium data in ${\text{H}}_{2}\text{O}-{\text{CO}}_{2}/{\text{H}}_{2}$ hydrates, Li et al. (2010) analyzed systems with 0.0014, 0.005 and 0.01 molar concentrations of TBAB promotor. The results indicated that by increasing the promotor concentration from 0.0014 to 0.005, the required pressure for hydrate formation was decreased. However, further increase from 0.005 to 0.01 worsened the situation, an intriguing observation that suggests the existence of an optimum promotor concentration for each system.

Wang et al. (Wang et al., 2015) and Yang et al. (Yang et al., 2013) studied the importance of promotor type for composite hydrates by comparing the hydrate formation condition for ${\text{CH}}_{4}$ and THF/SDS as the promotors in a composite ${\text{H}}_{2}\text{O}-{\text{CO}}_{2}/{\text{H}}_{2}$ hydrate system. The results showed that THF/SDS promotor significantly facilitates hydrogen storage by allowing hydrate formation to take place at more moderate conditions compared with those required when ${\text{CH}}_{4}$ is used. Also, temperature had considerable effect on hydrate formation for this case, where an increase in the temperature from 274 K to 282 K resulted in a rise in the required pressure from 5.5 MPa to 13.7 MPa.

#### Kinetics of hydrate formation

**Figure 6:**
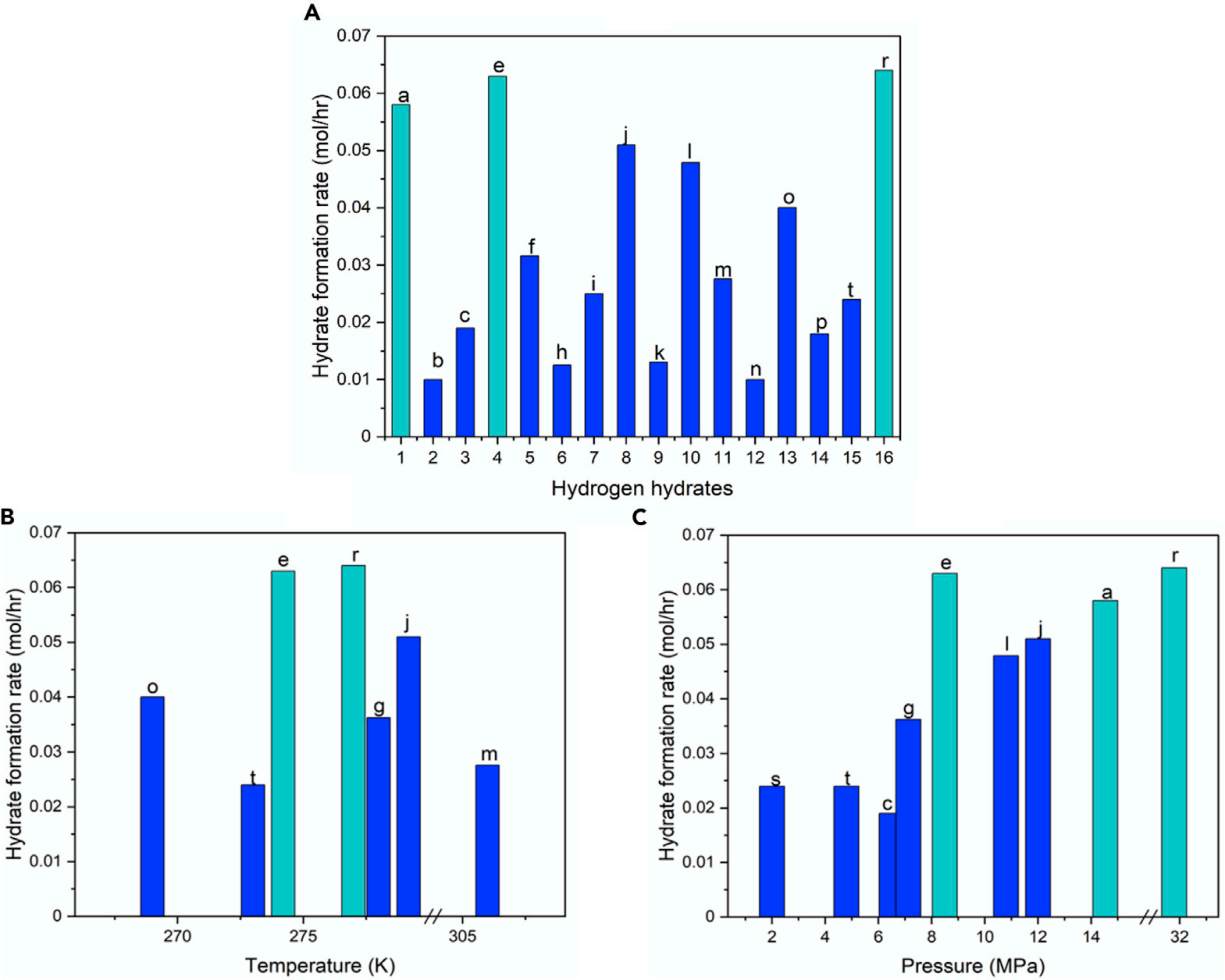
Kinetics of hydrogen hydrate formation Reported hydrogen formation rate for different hydrogen hydrate promotors (A). Reported hydrogen formation rate for different operating temperatures (B). Reported hydrogen formation at a range of operating pressure (C). Note that some bars overlap with one another and only highest measured value at each thermodynamic condition is reported. The complete dataset is given in Table 1. The labels on the bars correspond to the labels in Table 1.

The ${\text{H}}_{2}$ charging time is one of the critical factors for hydrogen storage applications. The charging time depends on the kinetics of hydrate formation and can be characterized by hydrate formation rate (mol/h). Although we discussed the importance of forming hydrogen hydrate at moderate pressures and temperatures, reasonable hydrate formation rate is important, as well. The hydrogen hydrate formation rate depends on different parameters including operating temperature, pressure, type of promotor, and promotor concentration. Table 1 and Figure 6A show the reported values of hydrogen hydrate formation rate from several recent studies carried out with different operating conditions i.e., pressure, temperature, promotor type, and promotor concentration. Among different parameters, many researches have studied the effect of promotor type on formation rate to identify the promotors that benefit the kinetics of hydrogen hydrate formation the most (Veluswamy et al., 2014b). THF has been shown to be one of the best promotors to enhance the kinetics of hydrate formation. For example, as shown in Figure 6B, Ogata et al. (Ogata et al., 2008) successfully formed hydrate with a very high formation rate at 277 K and 31.8 MPa by adding THF as the promotor.

**Table 1:** Formation rate of hydrogen hydrates with different promotors^a^

Promotor	Promotor concentration (Mol%)	Temperature (K)	Pressure (MPa)	Formation rate (mol/hr)	Label	Reference
THF	3	273.15	14.53	0.058	a	(Cai et al., 2019)
${\text{C}}_{3}{\text{H}}_{8}$	9.5	274.2	4.5	0.010	b	(Veluswamy et al., 2014c)
${\text{C}}_{3}{\text{H}}_{8}$	9.5	274.2	6.5	0.019	c	
${\text{C}}_{3}{\text{H}}_{8}$+SDS	9.5 + 100ppm	274.2	8.5	0.063	e	
THF + DTAC	5.6 + 0.0	278	7.13	0.0316	f	(Veluswamy et al., 2015a)
THF + DTAC	5.6 + 0.5	278	7.13	0.0362	g	
C_3_H_8_	9.5	277.2	4.5	0.0125	h	(Veluswamy, et al., 2015b)
C_3_H_8_	9.5	274.2	8.5	0.0250	i	
THF	3.5	279.2	12	0.051	j	(Veluswamy et al., 2014a)
TBAB	3.5	279.2	12	0.013	k	
THF	5	278.2	10.8	0.0479	l	(Veluswamy and Linga, 2013)
TBAF	3.4	306	5	0.0276	m	(Trueba et al., 2013)
TBAF	1.8	306	5	0.01	n	
TBAB	2.6	269	4.02	0.04	o	(Komatsu et al., 2013)
C_3_H_8_	2.5	273	3.5	0.018	p	(Kumar et al., 2009)
C_3_H_8_	1.2	273	4.8	0.024	t	
THF	0.8	277	31.8	0.064	r	(Ogata et al., 2008)
DSS	0.34	274	2	0.024	s	(Farhadi and Mohebbi, 2017)

a The labels in the table correspond to the bars in Figure 6.

The role of other parameters such as pressure and temperature in hydrate formation rate has been investigated, as well. Several studies have monitored the formation rate variation by changing a single parameter and maintaining other conditions. Veluswamy et al. (Veluswamy et al., 2014c, 2015a) showed that a decrease in pressure (while other conditions i.e., promotor type, promotor concentration, and temperature are fixed) adversely affected the hydrate formation rate. In fact, by decreasing the pressure from 8.5 MPa to 4.5 MPa, the formation rate dropped from 0.025 to 0.01 mol/h.

Sometimes the variation in formation rate due to simultaneous change in multiple parameters has been investigated. This is especially useful due to the potential interconnection between some of the parameters affecting the formation rate, in which case the behavior observed from isolated variation in one parameter at a specific condition may not reflect the system behavior while other conditions vary. For example, every promotor works the best at specific temperatures or pressures and variation in temperature and/or pressure of the system can significantly affect the effectiveness of the promotor; hence, not a single promotor can be recommended for all conditions. Cai et al. (Cai et al., 2019) and Veluswamy et al. (Veluswamy and Linga, 2013) studied the effect of simultaneous change in temperature and pressure in a system with THF promotor. They reported that decreasing the temperature from 278 K to 273 K while increasing the pressure from 10.8 MPa to 14.3 MPa resulted in an overall enhancement of 21% in the formation rate. The general improvement in hydrate formation rate by increasing the pressure is evident in Figure 6C, for a range of promotors. Here, we further underscore the point that hydrate formation rate is not a sole function of pressure. In fact, although the formation rate depends on pressure, it is the accumulative effect of all parameters that determines the final formation rate. Hence, although a pressure increase is anticipated to improve the formation rate, its effect may be nulled or outweighed by potential impacts from other parameters such as an increase in the temperature.

Promotor concentration is one of the parameters that can be tuned to improve hydrate formation rate. Trueba et al. (Trueba et al., 2013) measured the hydrate formation rates for both 1.8% and 3.4% molar concentrations of TBAF promotor at the pressure and temperature of 5 MPa and 306 K, respectively. The increase in promotor concentration was found to effectively enhance the hydrate formation rate from 0.0100 to 0.0276 mol/h. On the other hand, studying the variations in formation rate in response to simultaneous change in pressure and promotor concentration is useful to recognize the relative significance of each of these parameters. Kumar et al. (Kumar et al., 2009) used propane as the promotor and studied the effects of pressure and promotor concentration on the formation rate. The experiments were carried out at 273K and showed that an increase in the pressure (from 3.5 MPa to 4.8 MPa) increases the formation rate even though the promotor concentration was decreased from 2.5 to 1.2. These observations can be used to demonstrate the dominance of pressure compared with the promotor concentration when propane is used as the promotor. However, these conclusions cannot, typically, be generalized as the results are prone to change with a different promotor or under different system conditions.

The effect of simultaneous change in temperature and pressure on the formation rate has been subject of several studies such as those conducted by Veluswamy et al. (Veluswamy et al., 2014a) and Kumasto et al. (Komatsu et al., 2013). In these studies, TBAB promotor was used to form hydrogen hydrate at two different operating conditions: case 1 with a temperature and pressure of 279 K and 12 MPa, respectively and case 2 at 269 K and 4 MPa. Hydrogen hydrate formation was slower in case 1, despite it was carried out under a significantly higher pressure. Thus, temperature is believed to play a more important role in determining the kinetics of hydrate formation with TBAB.

Different promotors impact the hydrate formation rate to different extents. Veluswamy et al. (Veluswamy et al., 2014a), for example, compared the performances of two promotors, i.e., THF and TBAB, with identical concentration of 3.5% and under the same pressure and temperature conditions of 12 MPa and 279.2 K, respectively. Faster hydrate formation was observed with THF that signals its superior performance compared with TBAB. The addition of surfactant to the promotor is yet another effective way to enhance the kinetics of hydrate formation. Veluswamy et al. (Veluswamy et al., 2015a) showed that the addition of 0.5% dodecyl trimethyl ammonium chloride (DTAC) to a system with THF promotor improves the formation rate from 0.0316 to 0.0362 mol/h at otherwise identical conditions. Given the large range of promotors that can be added to a system and temperatures and pressures under which hydrate formation can take place, there is still room for scientific research to identify the optimum conditions for fast hydrate formation.

### Hydrogen hydrate dissociation

As stated earlier, one of the most significant advantages of application of hydrate systems for hydrogen storage is the fast and simple, on-demand release of hydrogen. In fact, in clathrate structure, hydrogen is stored in molecular form. Hence, no chemical reaction is required for the hydrogen release and the binding energy is small, thus eliminating the possibility of excessive heat required for the decomposition. Molecular hydrogen can be easily recovered from the hydrate through depressurization, thermal stimulation, or a combination of both approaches.

Cai et al. (Cai et al., 2019) compared the hydrate dissociation process in ${\text{H}}_{2}\text{O}-{\text{H}}_{2}/\text{THF}$ hydrate systems prepared in fresh water and memory water. The results indicated that memory effect has no significant influence on thermal state of ${\text{H}}_{2}\text{O}-{\text{H}}_{2}/\text{THF}$ hydrate. They also found an increasing trend in dissociation temperature by increasing the pressure. For example, they reported hydrate dissociation temperature of 282.41 K, 284.09 K and 285.82 K at the pressure of 18.00 MPa, 25.00 MPa, and 34.00 MPa, respectively.

Kinetics of hydrate dissociation is of paramount importance for different applications, because it determines the highest rate at which hydrogen can be supplied. Veluswamy et al. (Veluswamy et al., 2015a) realized that stirring significantly improves the decomposition kinetics in ${\text{H}}_{2}\text{O}-{\text{H}}_{2}/\text{THF}$ hydrate system. On the other hand, addition of surfactant showed no influence on decomposition kinetics for experiments carried out both with and without stirring. In a separate study, Veluswamy et al. (Veluswamy et al., 2015b) investigated the decomposition in hydrogen/propane mixed gas hydrate. The required heat for dissociation appeared to increase for mixtures with higher mole fractions of propane. A decrease in dissociation rate was observed for systems with higher propane content, as well. Thus, despite its positive role in facilitating the hydrate formation process, propane seems to adversely impact the hydrogen discharge properties of a hydrate system.

## Figures of merit

Hydrogen storage through clathrate hydrate formation attracted substantial attention as soon as its possibility was confirmed both experimentally and through simulations. However, as a relatively new technology, it must meet certain criteria to be utilized for different applications requiring hydrogen supply. Here, we discuss figures of merit that need to be heeded for hydrogen hydrate systems to make them a viable candidate for such applications. Because many applications rely on pure ${\text{H}}_{2}$ supply, achieving highly purified ${\text{H}}_{2}$ is of critical importance. Hence, separation efficiency can be considered an important figure of merit. In addition, one of the most obvious characteristics of any storage technique is its storage capacity. Therefore, we define hydrate storage capacity as another figure of merit, whereas especial attention needs to be paid to ensure that storage can take place at more moderate conditions.

### Hydrogen purification

**Figure 7:**
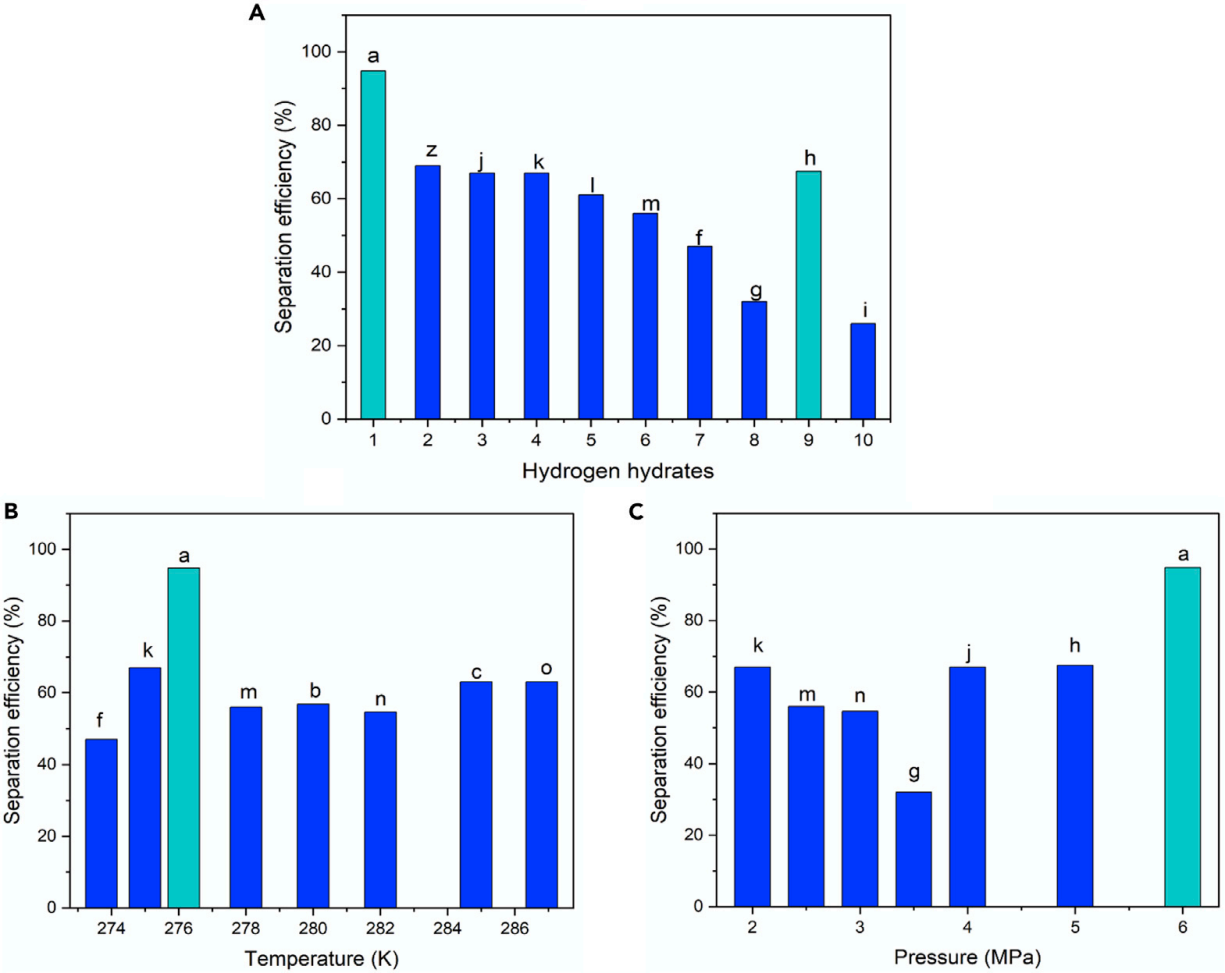
Hydrogen separation efficiencies Effect of promotor on separation efficiency in ${\text{H}}_{2}\text{O}-{\text{H}}_{2}$/${\text{CO}}_{2}$ hydrate system (A). Note that each bar corresponds to specific pressure and temperature at which hydrate formation was performed. Also, separation efficiency for different operating temperatures (B). Separation efficiency for different operating pressures (C). Note that some bars overlap with one another and only highest measured value at each thermodynamic condition is reported. The complete dataset is given in Table 2. The labels on the bars correspond to the labels in Table 2.

The main figure of merit for ${\text{H}}_{2}\;$purification is the separation efficiency. Separation efficiency depends on properties such as promotor type, temperature, and pressure, and different studies carried out under different conditions have reported a variety of efficiencies as reported in Table 2. Figure 7 compares the separation efficiency in different studies. Yu et al. (Yu et al., 2020b) were able to reach a separation efficiency of 98% by using cyclopentane (CP) as the promotor, which shows the potential of hydrate-based method as an efficient way for ${\text{H}}_{2}$ purification. Achieving high separation efficiencies for hydrogen purification at relatively low pressures is strongly desired.

**Table 2:** Separation efficiency of $CO_{2}$ from $H_{2}/CO_{2}\;$ mixture through hydrate formation^a^

Promotor	Promotor concentration (Mol%)	Temperature (K)	Pressure (MPa)	Separation efficiency	Label	Reference
CP	0.	276.15	6	90.9	a	(Yu et al., 2020b)
CP	1.33	276.15	6	94.8		
CP	0.34	276.15	6	98.8		
TBAB TBAB TBAB	0.29 0.5 1	278.15 280.35 282	3 3 3	23.8 56.88 54.63	b n	(Li et al., 2011)
THF (fresh-water) THF (memory-water) THF (memory-water)	5.56 5.56 5.56	284.85 286.8 285.85	6 6 6	63 63 69	c o z	(Li et al., 2019)
TBAB TBAB TBAB/DMSO TBAB/DMSO	0.4 0.256 0.29/0.71 0.4/0.071	285.95 286.15 277.15 285.25	2.51 2.5 2.5 2.5	46 52 68 63		(Xia et al., 2016)
TBAB	0.29	275.15	3.5	64	e	(Xu et al., 2013)
${\text{C}}_{3}{\text{H}}_{8}$ ${\text{C}}_{3}{\text{H}}_{8}$	2.5 1.2	273.7 273.7	3.8 3.5	47 32	f g	(Babu et al., 2013)
TBAB TBAB	0.29 0.29	275 284	5 3	67.5 26	h i	(Yu et al., 2018)
None		277.15	4	67	j	(Yang et al., 2015)
${\text{TBANO}}_{3}$ TBPB	2 3.1	275 275	2 2	67 61	k l	(Fukumoto et al., 2015)
TBAB	0.1	278	2.5	56	m	(Horii and Ohmura, 2018)

a The labels in the table correspond to the bars in Figure 7.

Studies on the effect of promotor concentration showed that increase in concentration has positive effect on the purification efficiency. Babu et al. (Babu et al., 2013) was able to enhance the efficiency from 32% to 47% by increasing the concentration of promotor (propane in this case) from 1.2 to 2.5 mol%. Nevertheless, excessively high promotor concentrations have shown to adversely affect the separation efficiency. Hence, there seems to be an optimum promotor concentration that yields the maximum efficiency. In fact, the increase in promotor concentration leads to higher number of cages to be occupied by the promotor and a consequent decrease in separation efficiency. Li et al. (Li et al., 2011) measured the separation efficiencies obtained from 0.29%, 0.5%, and 1% concentration of TBAB promotor, where the highest separation efficiency was achieved with a 0.5% concentration of TBAB. Yu et al. (Yu et al., 2020b) also studied the concentration effect on separation efficiency using CP as the promotor at temperature and pressure of 276 K and 6 MPa, respectively. The results indicated that maximum efficiency was obtained with a 0.34% concentration of CP, whereas 0.3% and 1.33% CP concentrations led to lower efficiencies. Separation efficiency can also be improved through the addition of appropriate amounts of surfactant to the promotor. Xia et al. (Xia et al., 2016) improved the separation efficiency from 46% to 63% through the addition of dimethyl sulfoxide (DMSO) to TBAB promotor while keeping all other conditions intact.

It is speculated that the use of memory water may be beneficial for separation applications. Li et al. (Li et al., 2019) investigated the hydrogen purification using THF promotor with both fresh and memory water. It was shown that at an identical pressure of 6 MPa, a separation efficiency of 63% was achieved at temperatures of 284.8 K and 286.8 K for fresh water and memory water, respectively. Achieving the same efficiency while operating at higher temperatures indicates the improved separation performance with memory water. Also, decreasing the temperature of the memory water to the lower value of 285.85 led to efficiency enhancement to 69%.

Xu et al. (Xu et al., 2013) and Yu et al. (Yu et al., 2018) studied the changes in hydrogen separation efficiency for various pressures and temperatures while using TBAB as the promotor. The results showed that temperature decrement considerably enhances the separation efficiency: while pressure was fixed at approximately 3 MPa, the efficiency increased from 26% to 64%, only by decreasing the temperature from 284 K to 275 K. Also, increase of pressure showed positive effect on the efficiency.

It is clear that besides promotor concentration, the promotor type itself plays a major role in hydrogen purification and the careful choice of promotor can significantly enhance the separation efficiency. For example, Fukumoto et al. (Fukumoto et al., 2015) demonstrated that separation efficiency can be improved from 61% to 67% by switching from TBPB promotor to ${\text{ TBANO}}_{3}$, while all other conditions, i.e., pressure and temperature, remain the same at 2 MPa and 275K, respectively.

### Hydrogen storage capacity

**Figure 8:**
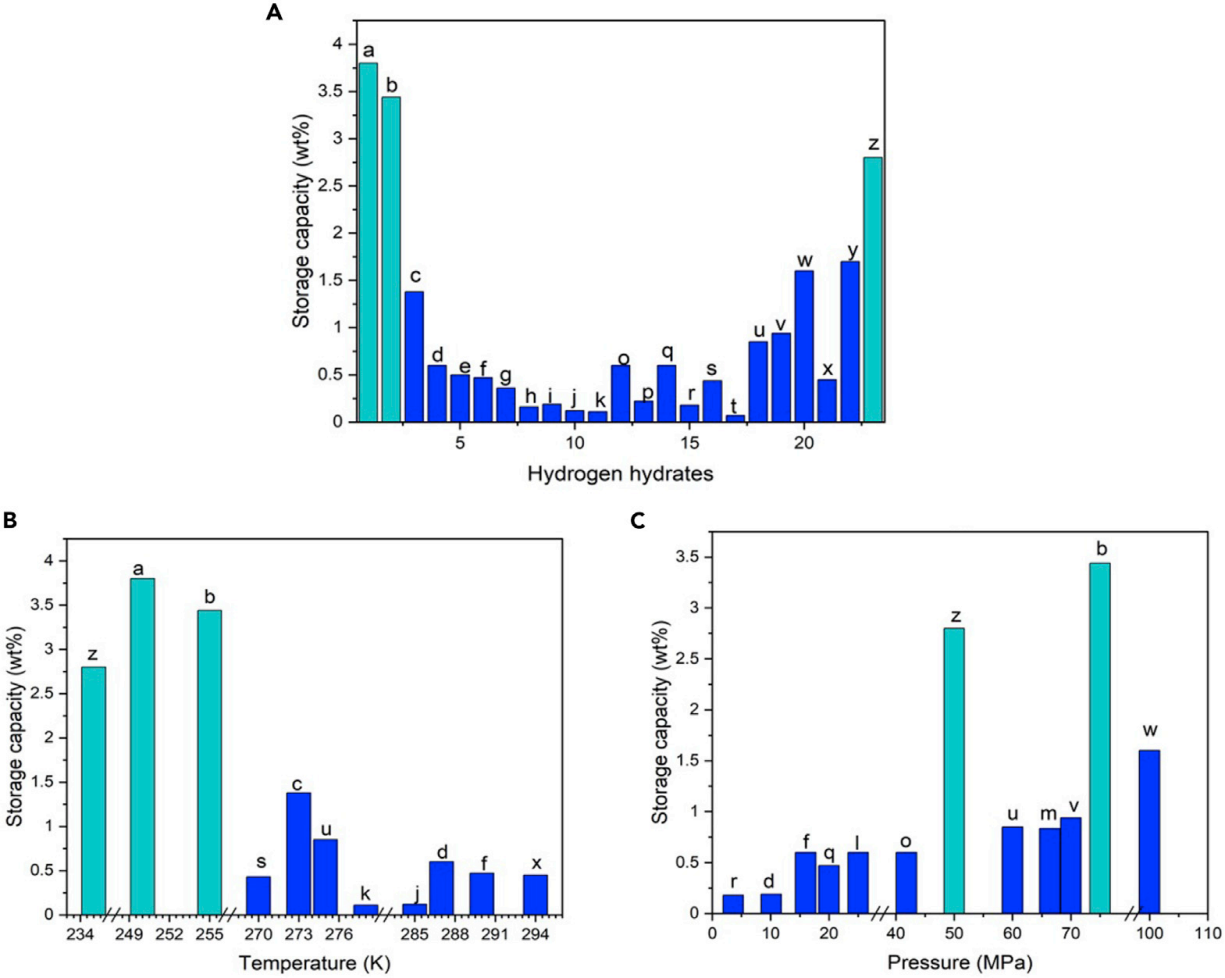
Hydrogen storage capacities Reported storage capacity for systems with different hydrogen hydrate promotors (A). Reported hydrogen storage capacity for different operating temperatures (B). Reported hydrogen storage for different operation pressures (C). Note that some bars overlap on top of each other and only highest measured value at each thermodynamic condition is reported. The complete dataset is given in Table 3. The labels on the bars correspond to the labels in Table 3.

The storage capacity is most important characteristic of any hydrogen hydrate storage material. Table 3 shows the storage capacities obtained in various studies employing different promotors and/or conducted at different temperatures and pressures. These storage capacities are also plotted in Figure 8. For hydrogen hydrate storage applications, it is desirable to achieve high storage capacities at relatively low pressures. As shown in Figure 8A, Zhdanov et al. (Zhdanov et al., 2019) obtained the highest storage capacity of 4.2% without any promotor. In general, it is possible to reach higher storage capacities without any promotor because the promotor itself occupies a portion of the empty cages and limits the available space for storing hydrogen molecules. However, as discussed previously, hydrogen hydrate formation without any promotor requires very high pressures or very low temperature, which are not desirable. Therefore, there is a trade-off between the storage capacity and achieving the desirable pressure and temperature. As demonstrated in Figures 8B and 8C, increased pressure and decreased temperature generally boost the storage capacity of the hydrogen hydrate method.

**Table 3:** Storage capacity of hydrogen hydrate for different promotors^a^

Promotor	Promotor concentration (Mol%)	Operating temperature (K)	Operating pressure (MPa)	Hydrogen storage capacity (wt.%)	Label	Reference
THF	3	273.15	14.53	1.875	t	(Cai et al., 2019)
None		250 250	150 450	3.8 4.2	a	(Zhdanov et al., 2019)
CP DHF DXL THP THT THF	5.6 5.6 5.6 5.6 5.6 5.6	278.4 271.5 269.2 272.3 274.5 276.2	10 10 10 10 10 10	0.11 0.16 0.36 0.19 0.5 0.12	k h g e	(Di Profio et al., 2018)
THF THF/carbon THF THF/carbon THF THF/carbon	5 5/70g 5 5/70g 5 5/70g	274 274 274 274 274 274	6.4 6.4 7.4 7.4 8.4 8.4	0.026 0.041 0.033 0.048 0.037 0.082		(Fang et al., 2014)
TBAOH	0.0323	290	20	0.47	f	(Karimi et al., 2014)
THF TBAB	3.5 3.5	279.2 279.2	12 12	0.169 0.052	r	(Veluswamy et al., 2014a)
C_2_H_6_ C_2_H_6_ C_2_H_6_ C_2_H_6_ C_2_H_6_ C_2_H_6_	1 1 1 5 5 5	250 250 250 250 250 250	25 100 250 25 100 250	0.5 1.6 2.4 0.2 0.7 1.25	w	(Belosludov et al., 2012)
THT THT THT Furan Furan Furan	5 5 5 5 5 5	275.1 275.1 275.1 275.1 275.1 275.1	15.4 32 41.8 15.5 32 41.8	0.25 0.43 0.6 0.23 0.47 0.59	n o	(Tsuda et al., 2009)
TBAB TBAB TBAB	1 3 6	279.5 279.5 279.5	13.8 13.8 13.8	0.1 0.22 0.048	p	(Strobel et al., 2007b)
THF THF	2 5.6	270 270	13.8 13.8	0.43 0.438	s	(Strobel et al., 2006)
TBAF TBAF	3.4 1.8	294 294	10 10	0.45 0.34	x	(Trueba et al., 2013)
Ar Ar	1 0.5	235 235	25 50	1.7 2.8	y z	(Zhdanov et al., 2017)
MCH MCH	0.4 1.6	273 274	149 25	1.38 0.6	c q	(Papadimitriou et al., 2008)
${\text{TBPBH}}_{4}$ TBAOH	2 2.5	285 285	12.1 12.1	0.12 0.14	j i	(Dolotko et al., 2011)
THF	3	255	75	3.44	b	(Sugahara et al., 2010)
TBAB	4	287	16	0.6	d	(Strobel et al., 2009)
THF THF THF THF	5.6 5.6 5.6 5.6	277.15 277.15 277.15 277.15	31.9 10.1 40.5 66.4	0.51 0.19 0.615 0.835	l m	(Ogata et al., 2008)
THF	2	269.5	3.6	0.18	r	(Nagai et al., 2008)
TBABh	4	100	0.1	0.07	t	(Shin et al., 2009)
DMCH MTBE	3 5	275 273	60 70	0.85 0.94	u v	(Martín and Peters, 2009)

a The labels in the table correspond to the bars in Figure 8.

Studying the effect of promotor type on storage capacity is crucial because promotor is recognized as an indispensable component in hydrate systems that can function in moderate conditions. Di Profio et al. (Di Profio et al., 2018) investigated the effect of promotor type on storage capacity by examining the obtainable storage with different promotors including CP, DHF, DXL, THP, THT, and THF under a fixed pressure of 10 MPa. It was shown that THT had the highest storage capacity of 0.5% among all the promotors. According to the results, the promotors can be sorted based on their impact on hydrogen storage as CP < THF < DHF < THP < DXL < THT, where THT and CP have demonstrated the lowest and highest negative impact on the storage capacity. Dolotko et al. (Dolotko et al., 2011) studied the hydrogen storage with two different promotors, i.e., tetra-n-butylphosphonium borohydride (${\text{TBPBH}}_{4}$) and tetra-n-butylammonium hydroxide (TBAOH). The results indicated that at a temperature and a pressure of 285K and 12MPa, respectively, both promotors led to almost identical storage capacities of 0.12% and 0.14%. It is found that the addition of activated carbon to promotors can enhance the storage capacity. Fang et al. (Fang et al., 2014) studied storage capacity for two types of promotors, i.e., pure THF and THF with activated carbon. They found that at an identical pressure and temperature of 6.4 MPa and 274 K, respectively, a storage capacity of 0.041% was obtained when using THF/activated carbon combination that was significantly higher than the 0.026% achieved via pure THF promotor. They also showed that storage capacity can be improved by increasing the pressure for both promotors.

Promotor concentration affects the storage capacity, as well. Strobel et al. (Strobel et al., 2006, 2007b) compared the storage capacities obtained with different concentrations of THF and TBAB. The results showed that an increase in promotor concentration enhances the storage capacity, but there exists an optimum value for the concentration after which the capacity fades. Veluswamy et al. (Veluswamy et al., 2014a) explored performance of THF and TBAB at temperature and pressure of 279 K and 12 MPa. It was also shown that storage capacity for the case with THF and TBAB were 0.169 and 0.052, respectively, which demonstrated better performance of THF for hydrogen storage enhancement.

As mentioned previously, temperature, pressure, and promotor type etc. are the parameters that strongly affect the storage capacity. Besides their individual direct influences on capacity, these parameters may be interacting with one another, leading to additional indirect influences on capacity. For example, the choice of promotor appears to depend on the working pressure, and there may not be a general promotor that works best at all conditions. A comparison between the capacities obtained from two different promotors i.e., THT and furan at three different pressures of 15.4 MPa, 32 MPa, and 41.8 MPa by Tsuda et al. (Tsuda et al., 2009) showed that THT results in higher capacities at pressures of 15.4 MPa and 41.8 MPa, whereas application of furan leads to higher capacity at 32 MPa. Furthermore, performance of different promotors under high pressures has been investigated. For example, in studies carried out by Papadimitriou et al.(Papadimitriou et al., 2008), Sugahara et al. (Sugahara et al., 2010), and Martín et al. (Martín and Peters, 2009), the hydrogen storage capacities obtained with different promotors, i.e., MCH, THF, MTBE, and DMCH, and at pressures of 145MPa, 75MPa, and 60MPa were measured. The following ranking of the promotors was presented according to the results: THF > MCH > MTBE > DMCH, which implies that THF with a storage capacity of 3.44% delivers the highest capacity.

Lower hydrogen storage capacity also implies larger amounts of water needed for hydrogen storage. For example, to store 1 gr of ${\text{H}}_{2}$ in a clathrate hydrate with storage capacity of 5 wt.%, about 20 mL of water is needed, whereas the required water volume can jump to ∼500 mL for a near-atmospheric pressure hydrate with a typical storage of 0.2 wt.%. However, storing water bulks in the order of several liters does not seem to pose any economic or environmental issues. Indeed, considering the high energy density of hydrogen gas, only few grams of ${\text{H}}_{2}$ can suffice for many applications. On the other hand, the water used for hydrogen storage is completely recyclable, i.e., it can be reused to form hydrate after its hydrogen content is released. Different studies have even shown slight improvements in formation rate when recycled water (memory water) is used. Thus, application of hydrate for hydrogen storage is further appealing as an economically justified and environmentally friendly technology.

## Future directions

Hydrogen clathrate hydrate is a highly promising medium as the storage material for ${\text{H}}_{2}$gas. This medium only contains water as the main crystal structure with immense environmental, economic, and technological benefits. However, the major challenges are the operating pressure and temperature range, low storage capacity, and low charging rate. Pure hydrogen hydrate can be formed at low temperatures and high pressure (i.e. 50–200 MPa) with storage capacity of 4.2 wt.%. The introduction of a promoter (e.g., THF) or mixture of promoters with few percentage concentrations could drastically ease the required operating condition for hydrate formation to ambient temperature and ambient pressure. However, the promoter molecules occupy a portion of empty cages in the hydrates structure, leading to sharp drop in the storage capacity of the hydrogen hydrate (i.e., approximately two orders of magnitude). Thus, there has been a wide range of studies to examine a range of promoters to achieve high storage capacity while having desired operating conditions. The promotors are ranked based on their role on hydrogen storage as CP < THF < DHF < THP < DXL < THT, where THT and CP have provided the maximum and minimum storage capacity. Furthermore, there is optimal concentration for these promoters to provide maximum storage capacity. The remaining challenge is to boost the storage capacity while keeping the desired operating condition. A combination of hydrogen hydrates and other storage mediums such as nano-porous carbons could provide shortcuts to achieve high storage capacity. We should add that due to high energy density of ${\text{H}}_{2}$ gas and low mass density of hydrogen hydrate (i.e. low weight), even 1% storage capacity could translate to future technologies. Further innovation and studies are required in long-cyclic performance of these storage medium, as consistent storage capacity is required for end-user application.

On the charging/discharging rate of ${\text{H}}_{2}\;$ in these mediums, there are more challenges to overcome. By nature, hydrogen hydrate formation rate is low and in order of 0.05 mol/h. THF has shown to be the most effective promoter to boost the formation rate. Let us consider a scenario for utilization of hydrogen hydrate as a battery in automobiles. The approximate energy use for the land transportation is 0.02 kWh/km. If a car drives on average 50 km per day, it requires 3600 kJ of energy, which corresponds to 2.5 grams of ${\text{H}}_{2}$gas. With storage capacity of 5 wt.%, this translates to 50 gr of required hydrogen hydrate per day. With the formation rate of 0.05 mol/h, the required time for charging the battery is approximately 54 h, which is quite high. Thus, immediate innovation to boost kinetics of hydrate formation is the demanding thrust at this time. Note that here we did not consider the efficiency of conversion of ${\text{H}}_{2}$ to the electrical/mechanical energy. The studies on the discharge rate are more limited, as it is a fast process and is not a bottleneck in utilization of hydrogen hydrate as future ${\text{H}}_{2}$ batteries. We think that research and innovation on material systems and kinetic of hydrate formation are the current open challenges.

## Conclusion

In this review, we provided a scientific and economical perceptive on critical role of hydrogen hydrates in transition and utilization of ${\text{H}}_{2}$ as the future fuel. Hydrogen hydrate as a medium for ${\text{H}}_{2}$ storage has a promising future in a wide spectrum of sectors, especially as a power source for automobiles, aircrafts, ships, and spacecrafts. The major role players in the hydrogen hydrate formation and storage process are temperature, pressure, promotor material, and promotor concentration. Although it is possible to store hydrogen at higher capacity values without using promotors, it will require harsh operating conditions, including pressure of more than 100 MPa or temperature of lower that 190 K. To charge and discharge ${\text{H}}_{2}$ through hydrogen hydrates at moderate conditions, promoters are required, but they drastically reduce the storage capacity. The effectiveness and adverse effects of promotors strongly depend on the operating conditions such as pressure, and optimal choice is dictated by the characteristics of the systems. However, in moderate conditions, THF has been recognized as the most effective and the most commonly used promotors for hydrogen hydrate storage. There exists an optimal concentration of the promoter resulting in maximum hydrate formation rates. This optimal concentration depends on the pressure, temperature, and type of the promotor. In addition to storage capacity, addition of promotors also shows positive effect on separation efficiency of ${\text{CO}}_{2}$ from ${\text{CO}}_{2}/{\text{H}}_{2}$ gas mixture. Through using CP as the promotor, it is possible to achieve separation efficiencies greater than 90%.

The current challenges are the storage capacity, the charging rate of ${\text{H}}_{2}$in this storage medium, and long cyclic performance, while the last two are the more demanding issues. As the formation kinetics of these storage media are low, long-time molecular dynamic simulations and innovation in material systems could provide better understanding on the above issues and smoothen the path for translation of this safe and highly promising storage technology.

## Footnotes

Supplemental Information can be found online at https://doi.org/10.1016/j.isci.2020.101907.
