# Supplementary material for: The potential of hydrogen hydrate as a future hydrogen storage medium
Source: iScience. 2020 Dec 9;24(1):101907. doi: 10.1016/j.isci.2020.101907 (PMC7770607; doi:10.1016/j.isci.2020.101907)
Supplement: Document S1. Table S1 [file mmc1.pdf]

iScience, Volume *24*

Supplemental Information

The potential of hydrogen hydrate as a future hydrogen storage medium

Ali Davoodabadi, Ashkan Mahmoudi, and Hadi Ghasemi

Table S1. Phase equilibrium data for coexistence of three phases of hydrogen gas, liquid hydrogen and hydrogen hydrate

Hydrate	Promotor	Promotor Concentration (mol)	Temperature (K)	Pressure (MPa)	Reference
H_2/H_2O	CO_2/CH_4	0.2998	275.8	3.63	(Smirnov and Stegailov, 2013)
		0.2998	277.6	4.49	
		0.2998	278.8	5.16	
		0.2998	280.6	6.46	
		0.2998	283.1	9.09	
		0.2998	284.5	11.07	
		0.1995	275.4	5.53	
		0.1995	276.3	6.13	
		0.1995	277.0	7.0	
		0.1995	279.2	8.84	
		0.1995	280.6	10.67	
		0.1995	282.4	13.71	
H_2/H_2O	C_3H_8	0.095	274.2	1.53	(Du *et al.*, 2011)
		0.095	275.2	2.41	
		0.095	276.2	3.15	
		0.095	277.2	3.83	
		0.095	278.2	4.81	
		0.095	279.2	6.34	
		0.095	280.2	7.73	
		0.095	280.9	8.90	
H_2/H_2O	Methylcyclohexane/CH_4	0.05/0.05	284.82	5.052	(Jacobson, Hujo and Molinero, 2009)
		0.05/0.05	287.87	7.552	
		0.05/0.05	289.53	10.05	
		0.035/0.005	291.04	12.5	
		0.035/0.005	272.79	10.048	
		0.035/0.005	274.72	13.049	
H_2/H_2O	THF	0.05	277.5	0.1	(Komatsu *et al.*, 2010)

		0.05	277.6	0.55	
		0.05	278.0	1.55	
		0.05	280.1	8.3	
		0.05	281.4	13.3	
H_2/H_2O	TBAB	0.035	285.4	0.13	(Komatsu *et al.*, 2010)
		0.035	285.9	2.19	
		0.035	286.3	6.05	
		0.035	287.2	13.4	
H_2/H_2O	$TBANO_3$	0.037	282.2	9.61	(Willow and Xantheas, 2012)
		0.037	283.1	15.83	
		0.037	283.7	21.39	
		0.037	284.6	25.93	
H_2/H_2O	SF_6	0.1	279.3	2	(Karimi, Dolotko and Dalmazzone, 2014)
		0.1	280.6	3.02	
		0.1	281.4	3.53	
		0.1	282.5	4.519	
		0.5	280.25	0.695	
		0.5	281.35	0.897	
		0.5	283.15	1.286	
H_2/H_2O	TBPB	0.026	281.9	0.11	(Sloan, 1998)
		0.026	282.73	4.44	
		0.026	284.37	13.3	
		0.026	289.0	50.7	
		0.026	295.94	164.6	
H_2/H_2O	THF		282.0	11	(Klapproth *et al.*, 2019)
			282.8	13.7	
			284.0	19	
			286.2	32	
H_2/H_2O	TBAB	0.006	279.4	0.52	(Liu *et al.*, 2017)
		0.006	280	3.11	
		0.006	281.3	11.5	
		0.006	283.8	0.49	
		0.02	283.87	1.52	

		0.02	285.9	13.7	
H_2/H_2O	THT	0.05	276	0.29	(Zhong *et al.*, 2020)
		0.05	277.9	5.86	
		0.05	278.9	8.88	
H_2/H_2O	Furan	0.05	277.3	0.11	(Zhong *et al.*, 2020)
		0.05	278.8	4.85	
		0.05	280.1	8.71	
H_2/H_2O	CO_2		271.5	1.52	(Hashimoto *et al.*, 2008)
			280.8	4.07	
			284.2	7.15	
H_2/H_2O	CO_2/ THF+SDS	0.01/1g	283.75	8.23	(Du *et al.*, 2012)
		0.01/1g	284,55	7.55	
		0.01/1g	282.45	4.57	
		0.01/1g	279.75	3.41	
		0.01/1g	278.5	3.0	
H_2/H_2O	CO_2/THF	0.6526/0.06	277.7	0.20	(Jacobson and Molinero, 2010)
		0.6526/0.06	282.6	0.68	
		0.6526/0.06	286.4	1.44	
		0.6526/0.06	288.4	1.89	
		0.2124/0.06	277.7	0.55	
		0.2124/0.06	283.2	1.96	
		0.2124/0.06	286.2	3.31	
		0.2124/0.06	288.2	4.46	
		0.0215/0.06	278.2	2.09	
		0.0215/0.06	281.2	6.31	
		0.0215/0.06	282.2	8.86	
H_2/H_2O	CO_2/TBAB	0.0014	275.15	0.51	(Koyama, Tanaka and Koga, 2005)
		0.0014	277.15	1.71	
		0.005	279.55	3.88	
		0.005	281.15	5.21	
		0.005	279.55	0.25	
		0.005	281.95	1.55	
		0.005	283.25	2.41	

		0.005	285.05	4.58	
		0.01	282.45	0.52	
		0.01	283.8	1.42	
		0.01	286.25	3.20	
H_2/H_2O	MTBE	0.012	269	70	(Liu *et al.*, 2019)
		0.012	270	76	
		0.012	272	100	
H_2/H_2O	DMCH	0.021	274.8	60	(Liu *et al.*, 2019)
		0.021	278	80	
		0.021	279.7	90	
H_2/H_2O	LN_2/THF	0.056	150	60	(Tsuda *et al.*, 2009)
		0.056	175	64	
		0.056	200	73	
H_2/H_2O	THF		280	8	(Patchkovskii and Tse, 2003)
			285	27	
			290	41	
H_2/H_2O			260	200	(de Menezes *et al.*, 2019)
			258	100	
			178	50	
H_2/H_2O	THF	0.053	278.3	2.09	(Lee *et al.*, 2016)
		0.053	280.4	8.03	
		0.053	282	12.96	
H_2/H_2O	CP	0.055	280.7	2.463	(Lee *et al.*, 2016)
		0.055	283.2	10.2	
		0.055	284.3	14	
H_2/H_2O	TBAOH	0.0083	286	1	(Khokhar, Gudmundsson and Sloan, 1998)
		0.0083	289	5.1	
		0.0083	290	20	
		0.0196	289	1.07	
		0.0196	292	10.08	
		0.0196	296	39	
H_2/H_2O	Ar	0.001	200	32	(Matsumoto and Tanaka, 2011)
		0.001	235	61	

		0.001	250	80	
		0.001	260	105	
		0.1	200	14	
		0.1	235	30.2	
		0.1	250	39.7	
		0.1	260	50	
		0.2998	275.8	3.63	
		0.2998	277.6	4.49	
		0.2998	278.8	5.16	
		0.2998	280.6	6.46	
		0.2998	283.1	9.09	
		0.2998	284.5	11.07	
H_2/H_2O	CO_2/CH_4	0.1995	275.4	5.53	(Smirnov and Stegailov, 2013)
		0.1995	276.3	6.13	
		0.1995	277	7.0	
		0.1995	279.2	8.84	
		0.1995	280.6,	10.67	
		0.1995	282.4	13.71	

REFERENCES*

Du, J. *et al.* (2012) 'Phase equilibria and dissociation enthalpies of hydrogen semi-clathrate hydrate with tetrabutyl ammonium nitrate', *Journal of Chemical and Engineering Data*, 57(2), pp. 603–609. doi: 10.1021/je201177t.

Du, J. W. *et al.* (2011) 'Hydrate phase equilibrium for the (hydrogen + tert-butylamine + water) system', *Journal of Chemical Thermodynamics*, 43(4), pp. 617–621. doi:

10.1016/j.jct.2010.11.018.
Hashimoto, S. *et al.* (2008) 'Thermodynamic stability of hydrogen + tetra-n-butyl ammonium bromide mixed gas hydrate in nonstoichiometric aqueous solutions', *Chemical Engineering Science*, 63(4), pp. 1092–1097. doi: 10.1016/j.ces.2007.11.001.
Jacobson, L. C., Hujo, W. and Molinero, V. (2009) 'Thermodynamic stability and growth of guest-free clathrate hydrates: A low-density crystal phase of water', *Journal of Physical Chemistry B*. American Chemical Society, 113(30), pp. 10298–10307. doi: 10.1021/jp903439a.
Jacobson, L. C. and Molinero, V. (2010) 'A methane-water model for coarse-grained simulations of solutions and clathrate hydrates', *Journal of Physical Chemistry B*. American Chemical Society, 114(21), pp. 7302–7311. doi: 10.1021/jp1013576.
Karimi, A. A., Dolotko, O. and Dalmazzone, D. (2014) 'Hydrate phase equilibria data and hydrogen storage capacity measurement of the system H2+tetrabutylammonium hydroxide+H2O', *Fluid Phase Equilibria*, 361, pp. 175–180. doi: 10.1016/j.fluid.2013.10.043.
Khokhar, A. A., Gudmundsson, J. S. and Sloan, E. D. (1998) 'Gas storage in structure H hydrates', *Fluid Phase Equilibria*. Elsevier Sci B.V., 150(151), pp. 383–392. doi: 10.1016/s0378-3812(98)00338-0.
Klapproth, A. *et al.* (2019) 'Kinetics of sII and Mixed sI/sII, Gas Hydrate Growth for a Methane/Propane Mixture Using Neutron Diffraction', *Journal of Physical Chemistry C*. American Chemical Society, pp. 2703–2715. doi: 10.1021/acs.jpcc.8b06693.
Komatsu, H. *et al.* (2010) 'Phase equilibrium measurements of hydrogen-tetrahydrofuran and hydrogen-cyclopentane binary clathrate hydrate systems', *Journal of Chemical and Engineering Data*, 55(6), pp. 2214–2218. doi: 10.1021/je900767h.
Koyama, Y., Tanaka, H. and Koga, K. (2005) 'On the thermodynamic stability and structural transition of clathrate hydrates', *Journal of Chemical Physics*. American Institute of PhysicsAIP, 122(7), p. 074503. doi: 10.1063/1.1850904.
Lee, Y. *et al.* (2016) 'Enclathration of CO2 as a co-guest of structure H hydrates and its implications for CO2 capture and sequestration', *Applied Energy*. Elsevier Ltd, 163, pp. 51–59. doi: 10.1016/j.apenergy.2015.11.009.
Liu, J. *et al.* (2017) 'Formation of clathrate cages of sI methane hydrate revealed by ab initio study', *Energy*, 120, pp. 698–704. doi: 10.1016/j.energy.2016.11.120.
Liu, J. *et al.* (2019) 'Prediction of efficient promoter molecules of sH hydrogen hydrate: An ab initio study', *Chemical Physics*. Elsevier B.V., 516, pp. 15–21. doi: 10.1016/j.chemphys.2018.08.032.
Matsumoto, M. and Tanaka, H. (2011) 'On the structure selectivity of clathrate hydrates', *Journal of Physical Chemistry B*. American Chemical Society, 115(25), pp. 8257–8265. doi: 10.1021/jp203478z.
de Menezes, D. É. S. *et al.* (2019) 'Coexistence of sI and sII in methane-propane hydrate former systems at high pressures', *Chemical Engineering Science*, 208. doi: 10.1016/j.ces.2019.08.007.
Patchkovskii, S. and Tse, J. S. (2003) 'Thermodynamic stability of hydrogen clathrates', *Proceedings of the National Academy of Sciences of the United States of America*. National Academy of Sciences, 100(25), pp. 14645–14650. doi: 10.1073/pnas.2430913100.
Sloan, E. D. (1998) 'Physical/chemical properties of gas hydrates and application to world margin stability and climatic change', *Geological Society Special Publication*. Geological Society of London, 137(1), pp. 31–50. doi: 10.1144/GSL.SP.1998.137.01.03.
Smirnov, G. S. and Stegailov, V. V. (2013) 'Toward determination of the new hydrogen

hydrate clathrate structures', *Journal of Physical Chemistry Letters*, 4(21), pp. 3560–3564. doi: 10.1021/jz401669d.
Tsuda, T. *et al.* (2009) 'Storage capacity of hydrogen in tetrahydrothiophene and furan clathrate hydrates', *Chemical Engineering Science*, 64(19), pp. 4150–4154. doi: 10.1016/j.ces.2009.06.018.
Willow, S. Y. and Xantheas, S. S. (2012) 'Enhancement of hydrogen storage capacity in hydrate lattices', *Chemical Physics Letters*, 525–526, pp. 13–18. doi: 10.1016/j.cplett.2011.12.036.
Zhong, H. *et al.* (2020) 'Two-dimensional Hydrogen Hydrate: Structure and Stability Molecular Dynamics Simulations of Inhibition Mechanism of Clay Swelling Inhibitors View project Two-dimensional hydrogen hydrates: structure and stability †', *Article in Physical Chemistry Chemical Physics*. doi: 10.1039/C9CP06296C.
